# Nonlinear dose-response of mind-body exercise in older adults with mild cognitive impairment: A systematic review and meta-analysis

**DOI:** 10.1371/journal.pone.0350867

**Published:** 2026-09-25

**Authors:** Yan-Fang Peng, Xi Chen, Guo-Qing Zhu, Guang-Cai Qu

**Affiliations:** 1 School of Physical Education, Wuhan Sports University, Wuhan, Hubei, China; 2 School of Sport Science, Wenzhou Medical University, Wenzhou, Zhejiang, China; 3 School of Rehabilitation Medicine, Wenzhou Medical University, Wenzhou, Zhejiang, China; 4 Graduate School of Wuhan Sports University, Wuhan, Hubei, China; University of Thessaly Faculty of Medicine: Panepistemio Thessalias Tmema Iatrikes, GREECE

## Abstract

**Objective:**

This study aimed to quantify domain-specific dose-response relationships between mind-body exercise and cognitive function in older adults with mild cognitive impairment (MCI), and to derive actionable dosing recommendation.

**Methods:**

Eight major electronic databases were systematically searched up to September 15, 2025. Randomized controlled trials (RCTs) of mind-body exercise intervention in individuals aged ≥60 years with MCI that reported at least one cognitive domain outcome were included. Hedges’g effect sizes were calculated using random-effects models, and dose-response relationships were examined using restricted cubic spline regression.

**Results:**

Twenty-seven RCTs (2034 participants) were included. Moderate-certainty evidence supported improvements in global cognition (Hedges’ g: 0.67, 95% CI: [0.49, 0.78]) and processing speed (0.45 [0.3, 0.6]). Global cognition exhibited a U-shaped weekly dose-response, with 240 min/week as the relative nadir that still yielded a significant pooled effect (0.37; [0.19, 0.55]); longer intervention durations significantly amplified this overall response (β = 0.012, p < 0.05), and achieving significant benefit at this dose required ≥11 weeks intervention. Memory followed a similar pattern, but with very low certainty. Processing speed showed a U-shaped cumulative dose-response (nadir: 3400 minutes), and this effect was attenuated by weekly dose (β = −0.0011, p < 0.05), yet caution is warranted because the weekly doses in the included studies were heavily clustered.

**Conclusions:**

Mind-body exercise produces domain-specific dose-response patterns in older adults with MCI. For global cognition, the observed U-shaped pattern suggests that if 240 min/week dose is adopted clinically, extending intervention to at least 11 weeks is advisable. However, prospective validation of these findings is needed before clinical application.

## Introduction

By 2050, the global prevalence of dementia is projected to increase nearly threefold to 139 million, demanding effective strategies against this progressive neurodegenerative disorder [1]. Mild cognitive impairment (MCI) is a delicate position between normal aging and early dementia, marked by both high risk and opportunity. Although the risk of progression to dementia is roughly tenfold higher [2], timely intervention can restore normal cognition in 14.4%−55.6% of cases [3].

Physical exercise has long been a cornerstone of non-pharmacological strategy to delay cognitive decline [4–7]. Among exercise modalities, mind–body exercises stand out as the most effective approach for improving cognition. A recent network meta-analysis of 61 RCTs (n = 5,458) demonstrated their superiority over six other non-pharmacological interventions for global cognition in old adults [8], and a larger analysis of 128 RCTs (n = 12,403) further confirmed their greater efficacy and higher adherence relative to conventional physical exercise in older individuals with cognitive impairment [9].

For MCI, global cognition benefits of mind-body exercise in MCI vary widely across studies, with substantial heterogeneous [10–13], and poorly characterized dose-response relationships. A recent study by Wen et al., [13] identified inverted U-shaped dose–response curves for multiple exercise parameters on global cognition, pinpointing an optimal regimen of three 45-minute sessions per week over 12–13 weeks. However, the available evidence exhibits substantial methodological heterogeneity (I^2^ ≈ 80%) and the certainty of evidence has not been assessed and reported. These limitations seriously restrict the formulation of accurate and unified mind-body exercise in intervention regimens for old adults with MCI. Meanwhile, two critical questions remain unclear: 1) whether the dose-response curves of a given exercise parameter is modulated by other parameters, and 2) whether the dose-response relationships observed for global cognition extend to other cognitive domains. To address these gaps, we applied restricted cubic spline regression to flexibly characterized domain-specific dose–response relationships between mind–body exercise and six cognitive domains in older adults with MCI, and to quantify interactions among program length (weeks), weekly dose (min/week), and cumulative dose (minutes).

## Methods

The study was pre-registered in PROSPERO (CRD42024562301) and performed following the PRISMA checklist [14]. The protocol is available at: PROSPERO (york.ac.uk).

### Search strategy

Eight databases including Pubmed, Embase, Scopus, Web of Science, Cochrane Library, China National Knowledge Infrastructure, Wanfang Data Knowledge Service Platform, and Chongqing VIP were systematically searched from inception to September 15, 2025. The detailed search strategy is provided in S1 Table in S1 File. Reference lists of eligible articles were also examined. Screening of titles, abstracts, and full tests was performed independently by two reviewers, with disagreements adjudicated by a third reviewer. The authors were unable to obtain information that could identify individual participants.

### Eligibility criteria

The studies were included if they are (1) randomized controlled trials, (2) written in English or Chinese, and (3) used any type of mind-body exercise as intervention. (4) Studies had to include a non-exercise controls; (5) had to enroll participants aged ≥ 60 years with clinically diagnosed MCI according to standardized criteria; and (6) had to reported at least one cognitive outcome (global cognition, memory, working memory, inhibition, flexibility, or processing speed). We excluded studies that (1) focused on the acute effects of mind-body exercise or combination it with other therapies; (2) enrolled participants whose MCI resulted from neurological trauma or structural brain abnormalities; (3) were duplicate publications with overlapping study samples; and (4) lacked sufficient outcome data, even after attempts to contact the authors. The selection of studies to be included was independently completed by two reviewers; disagreements were resolved through group discussion.

### Data extraction

Data were independently extracted by two reviewers (Zhu and Qu); disagreements were resolved through group discussion (with Peng and Chen). From each of the included studies, the following information was extracted: participant clinical diagnosis of MCI, age, intervention and control protocols, cognitive assessment tool, and all data required for effect size calculation at each time point. Following Cochrane guidance, we prioritized intention-to-treat (ITT) data over per-protocol results to preserve the benefits of randomization. When multiple tests per domain were available, the measure with higher cognitive demand was selected using predefined criteria [9]. In multi-arm trials, control group participants were distributed equally across comparisons, and unique study IDs were assigned to repeated measures. For time-based tasks, change scores were coded so that higher values consistently indicated better performance [15].

### Data coding and management

To minimize confounding between parameters, we operationalized mind-body exercise exposure using three key variables: weekly duration (minutes/week, calculated as frequency × duration per session), intervention duration (week), and cumulative duration (min, derived from weekly duration × intervention duration). Intensity was not considered, as the included interventions were homogeneous. For analysis, data were organized into four hierarchical levels: (1) six cognitive domains, including global cognition, memory, working memory, inhibition, flexibility, and processing speed; (2) weekly duration categorized as ≤150 min/week, 151–300 min/week, and >300 min/week; (3) intervention duration grouped into ≤8, ~ 12, ~ 16, ~ 24, and >24 weeks; and (4) cumulative duration stratified into ≤1440, 1441–2880, 2881–4320, 4321–5760, 5761–7200, and > 7200 minutes. Key study and intervention characteristics were tabulated for all included articles. Eligibility for each synthesis was determined by comparing the extracted study features against the pre-planned grouping categories.

### Data synthesis

The effect of mind-body exercise on each cognitive domain was estimated as Hedges’ g (g) with 95% confidence interval (CI) [15], using random-effects models applied to pre- and post-intervention change value. If change values were not reported, we computed mean change from baseline as:


$$\begin{array}{c} {Mean}_{change}=\;{Mean}_{post}-{Mean}_{pre} \end{array}$$ (1)

and Standard Deviation of the Change from baseline as:


$$\begin{array}{c} {\text{SD}}_{\text{change}}=\surd \left({\text{SD}}_{\text{post}}^{\text{2}}\right)+\left(SD_{pre}^{\text{2}}\right)-\left(\text{2}*Corr*SD_{post}*SD_{pre}\right) \end{array}$$ (2)

The pre-post correlation coefficient was set to r = 0.5 [16]. Magnitudes of effect sizes were defined as trivial (<0.2), small (0.2∼0.5), moderate (0.5∼0.8), or large (>0.8) [17].

Restricted cubic spline (RCS) regression, a flexible approach for modelling nonlinear associations [18], was employed to quantify the dose-response relationships between exercise parameters and domain-specific cognitive improvements. All available time-point data were included in the model fitting. Spline basis variables were generated using Stata 17.0’s mkspline command with the cubic option. The number of knots (3–5) was specified via the nknots (#) option, and knots were automatically placed at Harrell’s recommended percentiles of the dose variable distribution (e.g., for 4 knots: the 5th, 35th, 65th, and 95th percentiles) [19]. This percentile-based placement ensures that each knot is supported by adequate data density, preventing curve instability in data-sparse regions [20]. The exact knot positions for each model, as output by the displayknots option, are reported alongside the corresponding results in the Results section. We fitted a generalized estimating equation (GEE) model via xtgee incorporating restricted cubic spline terms, with an exchangeable working correlation structure specified for with-study correlation. Sandwich-type robust standard errors were clustered at the study level using the Stata option vce (cluster number). where number represented the unique study identifier. In the primary analysis, each included study was weighted equally to explore the basic nonlinear trend shape without allowing large-sample studies to disproportionately influence curve curvature. A sensitivity analysis using inverse-variance weighting (study-level mean of 1/SE^2^) was conducted to confirm that the observed curve shape was not dependent on the weighting scheme (S2 Table in S1 File). Model fit was compared using the Quasi-likelihood under Independence Criterion (QIC), with lower values indicating better fit. To identify the threshold at which statistical significance was first achieved, we determined the time point at which the 95% confidence interval of the effect estimate no longer crossed the null value. If the sample size was insufficient to support reliable knot generation for RCS, subgroup analyses stratified by predefined exercise parameters were conducted. All analyses were performed in Stata 17.0. The code is submitted as an attachment in S1 File.

### Risk of bias and quality of evidence

The studies quality was independently assessed and rated by three reviewers according to the Cochrane risk-of-bias tool, with each domain rated as high, low or unclear; any discrepancies were resolved through discussion. Publication bias was evaluated using funnel plots and Egger’s test [21], followed by the trim-and-fill adjustment (https://www.metaessentials.org/) to estimate an impute potentially missing studies [22], allowing comparison of effect sizes before and after correction. Heterogeneity was examined using Cochrane’s Q and quantified with I^2^; with significant heterogeneity defined as P < 0.10 and I^2^ > 50% [23]. Sensitivity analyses included subgroup stratification and sequential exclusion to test robustness.

### Certainty of evidence

The certainty of evidence for each outcome was assessed independently by two review authors (Peng and Zhu) using the Grading of Recommendations Assessment, Development and Evaluation (GRADE) framework (GRADE: an emerging consensus on rating quality of evidence and strength of recommendations) [24]. Disagreements were resolved through discussion or, when necessary, by consulting a third reviewer (Chen). The assessment was evaluated five domains. 1) Risk of bias was judged by evaluating key methodological aspects of the included trials, including randomization, allocation concealment, blinding, and handling of incomplete outcome date; 2) Inconsistency was quantified by the I^2^ statistic, with I^2^ < 50% considered not serious, 50–75% serious, and >75% very serious; 3) Indirectness was judged by the directness of the evidence in addressing the review question (PICO); 4) Imprecision was rated down if the total sample size was below the optimal information size of 400 participants for continuous outcomes, or if the 95% confidence interval crossed a minimal important difference of 0.2–0.5 for standardised mean differences [24]; 5) Publication bias (assessed by visual inspection of funnel plots for outcomes with at least 10 studies and by Egger’s regression test, with p < 0.1 considered suggestive of publication bias). Starting from high certainty for all included randomised controlled trials, each domain could be downgraded by one or two levels if judged as serious or very serious, respectively. The final certainty was rated as high (⊕⊕⊕⊕), moderate (⊕⊕⊕⊖), low (⊕⊕⊖⊖) or very low (⊕⊖⊖⊖). A summary of the GRADE assessments, including detailed reasons for downgrading, is presented in the Results section.

### Ethics statement

This is a meta-analysis of previously published studies. All data used in this analysis were extracted from published literature and are fully anonymized. Therefore, this study did not require ethic approval or informed consent.

## Results

### Study selection

**Fig 1:**
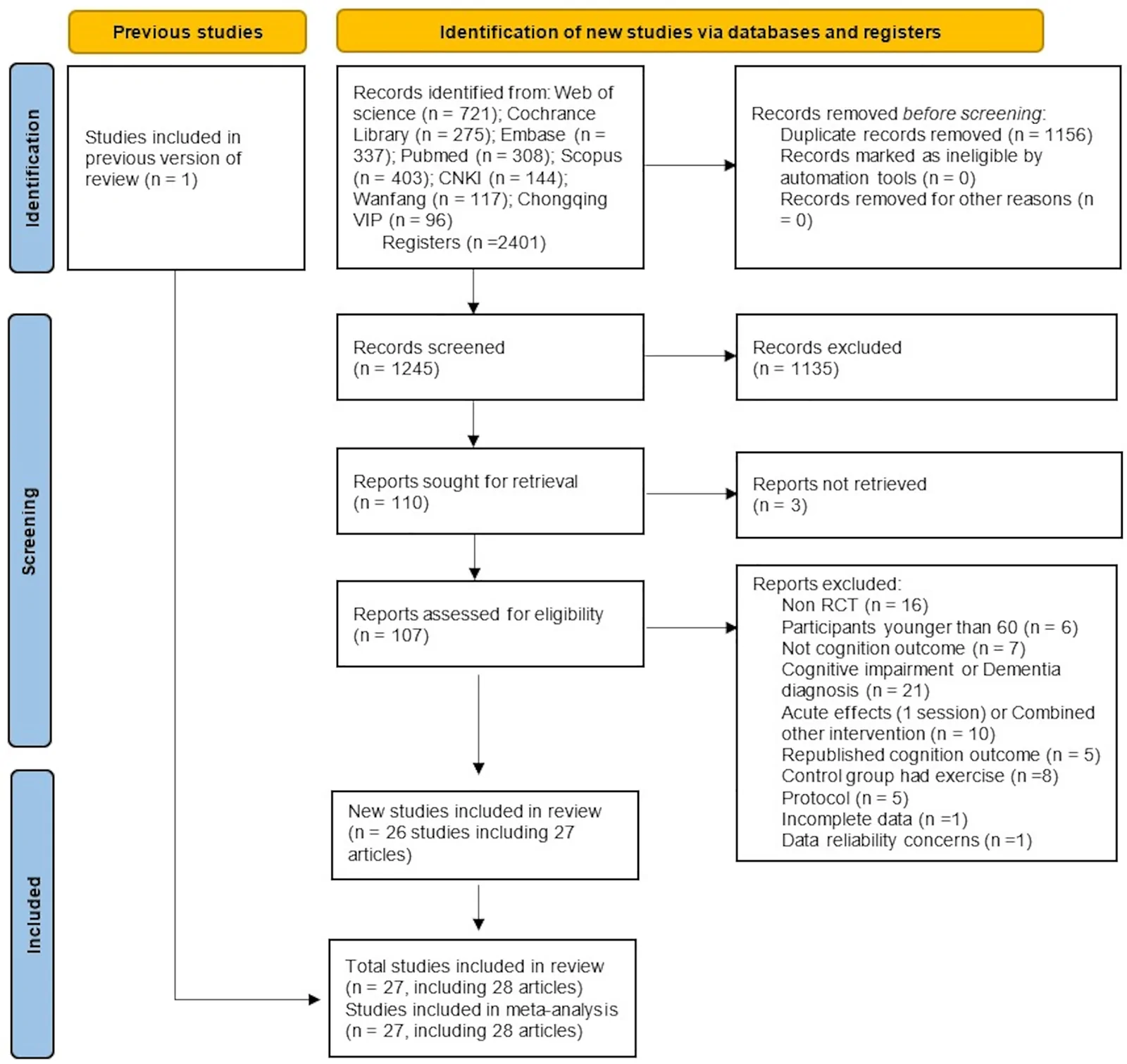
PRISMA flow diagram of study selection.

A total of 2401 records identified, 1,156 duplicates were removed, leaving 1,245 for title and abstract screening, from which 1,138 were excluded; the remaining 108 for full-text articles were assessed, yielding 28 RCTs that met the inclusion criteria. However, one study by Lin [25] reported a mean age of 67.13 (SD = 10.39) that was inconsistent with the stated age range of 60–73 years, raising concerns about data reliability. As a result, the final meta-analysis included 27 RCTs, reported across 28 articles (Fig 1).

### Description of included studies

This study included 27 RCTs involving 2056 older adults with MCI. Across these trials, 2034 participants (98.9%) were included in the data analyses, while 1,765 participants (85.8%) completed the full study protocol and provided valid data. Three studies employed a three-arm design with two mind-body exercise intervention groups, both of which met the eligibility criteria and one control group [26–28]. All experimental groups were analyzed independent against control group to assess their effects. Comorbid conditions were documented as follows: type II diabetes mellitus [29–31], hypertension [26], insomnia [32], and chronic obstructive pulmonary [33]. Due to mind-body exercise origins in traditional Chinese medicine, these studies primarily conducted in Chinese-speaking regions: 26 studies in mainland China, 2 in Taiwan China, one in Thailand (two publications). The literatures included 13 English articles, 10 Chinese articles, and 5 master’s theses from Chinese universities.

#### Intervention and control characteristics.

Across the studies, interventions lasted between 12 weeks and 1 year, with 24 weeks program being the most commonly implemented duration (n = 15). Seven trials incorporated interim assessments. Weekly exercise duration varied from 90 to 450 minutes, with 180 min/week the modal dose (n = 9). Cumulative intervention duration spanned from 800 to 10,800 minutes, with most programs delivering 1,800–4,320 minutes. No control groups adopted any regular exercise intervention, 14 studies incorporated education components as active controls.

#### Primary measured outcome.

Global cognition was evaluated in 22 studies, with 21 using the Montreal cognitive assessment (MoCA) [26–29,31–47], and 1 employing the Mattis Dementia Rating Scale (MDRS) [48]. Although the Mini-Mental State Examination (MMSE) was also assessed in five MoCA-based studies, the MoCA was selected as the primary for meta-analysis due to its superior sensitivity in detecting MCI [49]. Memory Function was assessed in 13 studies using the following measures: the Wechsler Memory Scale (WMS; n = 6) [35,37,44,45,50], the Logical Memory Test (n = 4) [28,34,51,52], the California Verbal Learning Test (CVLT) [27], and the Rivermead Behavioural Memory Test (RBMT) [41]. For meta-analysis, data on delayed recall, immediate recall, and total memory were separately extracted based on reported outcomes.

#### Secondary measured outcomes.

Secondary measured outcomes included 4 cognitive domains: inhibition, flexibility, working memory, and processing speed. Inhibition was measured in 6 studies using the Flanker task [53], Stroop task [27,28,54], and Go/No-Go task [44,55]. Flexibility was assessed in 3 studies, including the Shifting [53] and the Delta Trail Making Test (TMT, difference between part B and A) [27,51,52]. Working memory was evaluated in 7 studies: 5 employed the TMT-B [27,29,34,41,42], 1 used the 2-back task [53], and 1 (comprising two articles) applied the Digit Span Backward test [51,52]. Processing Speed was assessed in 9 studies, yielding 11 datasets: 6 studies used the TMT-A [27,28,34,35,41,50], 2 employed the Digit Symbol Substitution Test (DSST) [29,41], and 2 applied the Digit Symbol Coding Test (DSCT) [44,54] (Table 1).

**Table 1:** Characteristics of the included studies.

Author (year)	Region	Participants (C/E)	Intervention	Outcome/tool
Cai & Zhang (2018)	Hefei China	MCI N: 30/28 Age: 66.8(5.3)/67.5(6.3)	E: Mixed Qigong; 24 wk*; 5 times/wk; 90 min/time C: Usual activities	Global Cog: MoCA; MMSE MEM: LMT; DST WM: TMT-B PS: TMT-A
Chen et al. (2023)	Mul-center China	MCI-DM2 N: 111/107 Age: 67.6(5.6)/67.6(5.0)	E: TaiChi; 24 wk; 3 times/wk; 60 min/time C: No intervention	Global Cog: MoCA MEM: WMS WM: TMT-B PS: DSST
Guo (2018)	Shenyang China	MCI N: 15/15 Age: 63.3(3.1)/62.3(2.5)	E: Baduanjin; 16 wk; 5 times/wk; 60 min/time C: Usual activities	IC: go-nogo test
Hwang et al. (2023)	Taiwan China	MCI N: 63/63Age: ≥ 65	E: TaiChi; 24 wk; 3 times/wk; 50 min/time C: Health education	Global Cog: MDRS
Li (2016)	Fuzhou China	MCI N: 29/28 Age: 65.9(5.1)/66.6(4.0)	E: Baduanjin; 24 wk*; 3 times/wk; 60 min/time C: Health education	Global Cog: MoCA MEM: WMS; AVLT
Li (2018)	Shenyang China	MCI N: 15/15 Age: 62.1(2.1)/61.4(1.8)	E: Baduanjin; 16 wk; 5 times/wk; 40 min/time C: Usual activities	WK: 2-back taskI C: Flanker task CF: More-odd shifting task
Lin et al. (2024)	Fuzhou China	MCI N: 50/50 Age: 68.3(4.1)/67.4(3.9)	E: TaiChi; 12 wk; 5 times/wk; 60 min/time C: Health education	Global Cog: MoCA MEM: WMS
Lin et al. (2025)	Chengdu China	MCI N: 20/20 Age: 81.9(6.1)/86.2(6.2)	E: TaiChi; 12 wk; 3 times/wk; 90 min/time C: Usual activities	Global Cog: MoCA MEM: AVLT PS: TMT-A
Liu, Guo, & Bai (2018)	Taiwan China	MCI N: 29/28 Age: 71.6(5.3)/71.2(5.5)	E: Baduanjin; 24 wk; 6 times/wk; 60 min/time C: No intervention	Global Cog: MoCA
Liu et al. (2021)	Xian China	MCI N: 20/20 Age: 66.0(5.7)/66.2(4.2)	E: Baduanjin; 24 wk; 60 min/time; 3 times/wk C: Health education	Global Cog: MoCA
Liu et al. (2022)	Fuzhou China	MCI N: 18/18/18 Age: 73.4(6.5)/ 73.2(6.3)/74.6(6.1)	E1: TaiChi E2: VR-TaiChi 12 wk; 3 times/wk; 50 min/time C: Usual activities	Global Cog: MoCA MEM: CCVLT WK: TMT-BI C: Stroop test CF: TMT(B-A)
Ruan et al. (2024)	Fuzhou China	MCI-DM2 N: 33/35 Age: 68.6(5.2)/68.7(5.6)	E: TaiChi 24 wk; 3 times/wk; 60 min/time C: Health education	Global Cog: MoCA
Sun et al. (2021)	Nanjing China	MCI N: 30/30 Age: 65–85	E: VR Baduanjin 24 wk; 3 times/wk; 30 min/time C: Health education	Global Cog: MoCA MEM: RBMT WK: TMT-B PS: TMT-A; DSST
Sungkarat et al. (2016, 2018)	Chiang MaiThailand	MCI N: 33/33 Age: 67.5(7.3)/68.3(6.7)	E: TaiChi 24 wk*; 3 times/wk; 50 min/time C: Health education	MEM: LMDR WK: DST(f-b) CF: TMT (B-A)
Tian (2020)	Quanzhou China	MCI N: 55/55 Age: 65–85	E: TaiChi 16 wk*; 7 times/wk; 60 min/time C: Usual activities	Global Cog: MoCAWK: TMT-B
Wang (2018)	Shenyang China	MCI N: 15/15 Age: 63.6(3.4)/62.6(2.5)	E: Baduanjin 16 wk; 5 times/wk; 40 min/time C: No intervention	Global Cog: MoCA; MMSE
Xia et al. (2019)	Fuzhou China	MCI N: 23/23 Age: 65.9(5.3)/65.8(4.4)	E: Baduanjin 24 wk; 3 times/wk; 60 min/time C: Health education	IC: Stroop task PS: DSCT
Xia et al. (2023)	Fuzhou China	MCI N: 45/45 Age:65.4(4.9)/66.2(4.2)	E: Baduanjin 24 wk; 3 times/wk; 60 min/time C: Health education	Global Cog: MoCA MEM: WMS; AVLTCF: TMT-B/AI C: stroop test; go/nogo test PS: DSCT
Xu et al. (2023)	Shanghai China	aMCI N: 35/35 Age: 68.6(7.5)/67.5(7.3)	E: Baduanjin 12 wk; 3 times/wk; 50 min/time C: Health education	Global Cog: MoCA MEM: AVLT
Zhang et. al. (2020)	Changsha China	MCI N: 54/54 Age: 65–85	E: TaiChi 16 wk; 4 times/wk; 45 min/time C: Usual activities	MEM: AVLT PS: TMT-A
Yang et al. (2025)	Shanghai China	COPD-MCI N: 28/28 Age: 68.6(4.8)/69.2(4)	E: Baduanjin 12 wk; 3 times/wk; 90 min/week C: Routine care	Global Cog: MoCA
Zhang & Zhao (2024)	Beijing China	MCI-HTN N: 50/50/50 Age: 60–80	E1: Baduanjin E2: VR-Baduanjin 24 wk*; 3 times/wk; 60 min/time C: Health education	Global Cog: MoCA; MMSE MEM: WMS
Zheng et al. (2013)	Beijing China	aMCI N: 45/45 Age: 64.2(5.4)/65.3(5.3)	E: Liuzijue qigong 24 wk; 5 times/wk; 60 min/time C: No intervention	Global Cog: MoCA; MMSE
Zheng et al. (2021)	Fuzhou China	MCI N: 23/23 Age:65.9(5.3)/65.8(4.4)	E: Baduanjin 24 wk; 3 times/wk; 60 min/time C: Usual activities	Global Cog: MoCA MEM: WMS
Zhu et al. (2015)	Nanjing China	MCI-DM2 N: 43/43 Age: 69.9(6.4)	E: Baduanjin 52 wk*; 5 times/wk; 40 min/time C: Routine care	Global Cog: MoCA
Zhuang (2023)	Wuhan China	MCI-INS N: 31/30 Age: 71.7(2.8)/71.9(3.2)	E: Baduanjin 24 wk*; 7 times/wk; 180 min/wk C: Health education	Global Cog: MoCA
Zhou (2025)	multicenter China	MCI N: 22/22/22 Age: 66.9(4.9)/66.1(6.6)	E1: TaiChi E2: TaiChi with music 12 wk; 3 times/wk; 30 min/time C: Usual activities	Global Cog: MoCA; MMSE MEM: LMTI C: Stroop test PS: TMT-A

**Abbreviations:** aMCI: amnestic mild cognitive impairment; MCI-DM2: MCI with diabetes mellitus type 2; COPD-MCI: Chronic Obstructive Pulmonary Disease Patients with MCI; MCI-HTN: MCI with hypertension; MCI-INS: MCI with insomnia; C: control group; E: experimental group; *: at least one mid-intervention test; Global Cog: Global Cognition; MEM: Memory; WM: Working Memory; IC: Inhibition Control; CF: Cognitive Flexibility; PS: Processing Speed.

### Risk of bias of included studies

**Fig 2:**
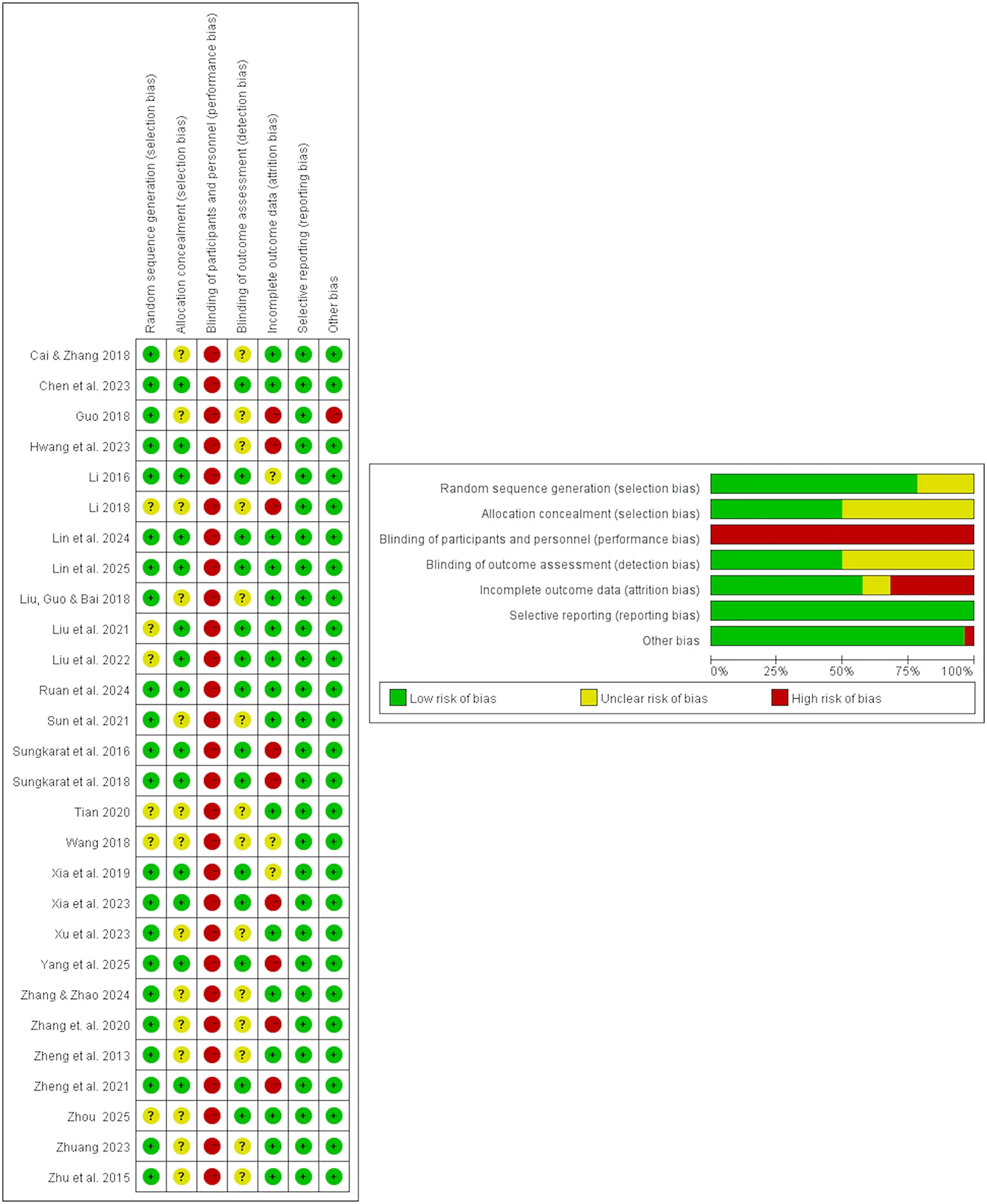
Risk of bias of included studies. Left panel represents risk of bias graph of included study; right panel represents risk of bias summary of included study.

All included studies were RCTs. Among them, 23 studies (85.2%) adequately described methods for random sequence generation and were rated as low risk of bias. However, only 13 studies (48.1%) explicitly reported allocation concealment, while the remaining 14 (51.9%) provided insufficient details and were thus classified as unclear risk. Due to the inherent challenges in blinding participants and personnel in behavioral interventions, all studies were assessed as having a high risk of bias in this domain. Regarding outcome assessment, 14 studies (51.9%) clearly implemented blinding procedures and rated as low risk. In terms of attrition bias, 7 studies (25.9%) exhibited a high risk due to data loss exceeding 10%, reporting reasons for dropout. Another 3 studies (11.1%) raised some concerns, as dropout rates were accepted but the causes of participant withdrawal were incompletely described. Whereas the remaining studies demonstrated acceptable retention rates and were considered low risk. Notably, all studies showed consistency between reported results and their original study designs, indicating low risk of reporting bias. One exception was the study by Guo [55] rated at high risk of other biases, driven by its small sample size (n = 12) and substantial data attrition (60%) (Fig 2).

### The effect of mind-body exercise on cognition

**Fig 3:**
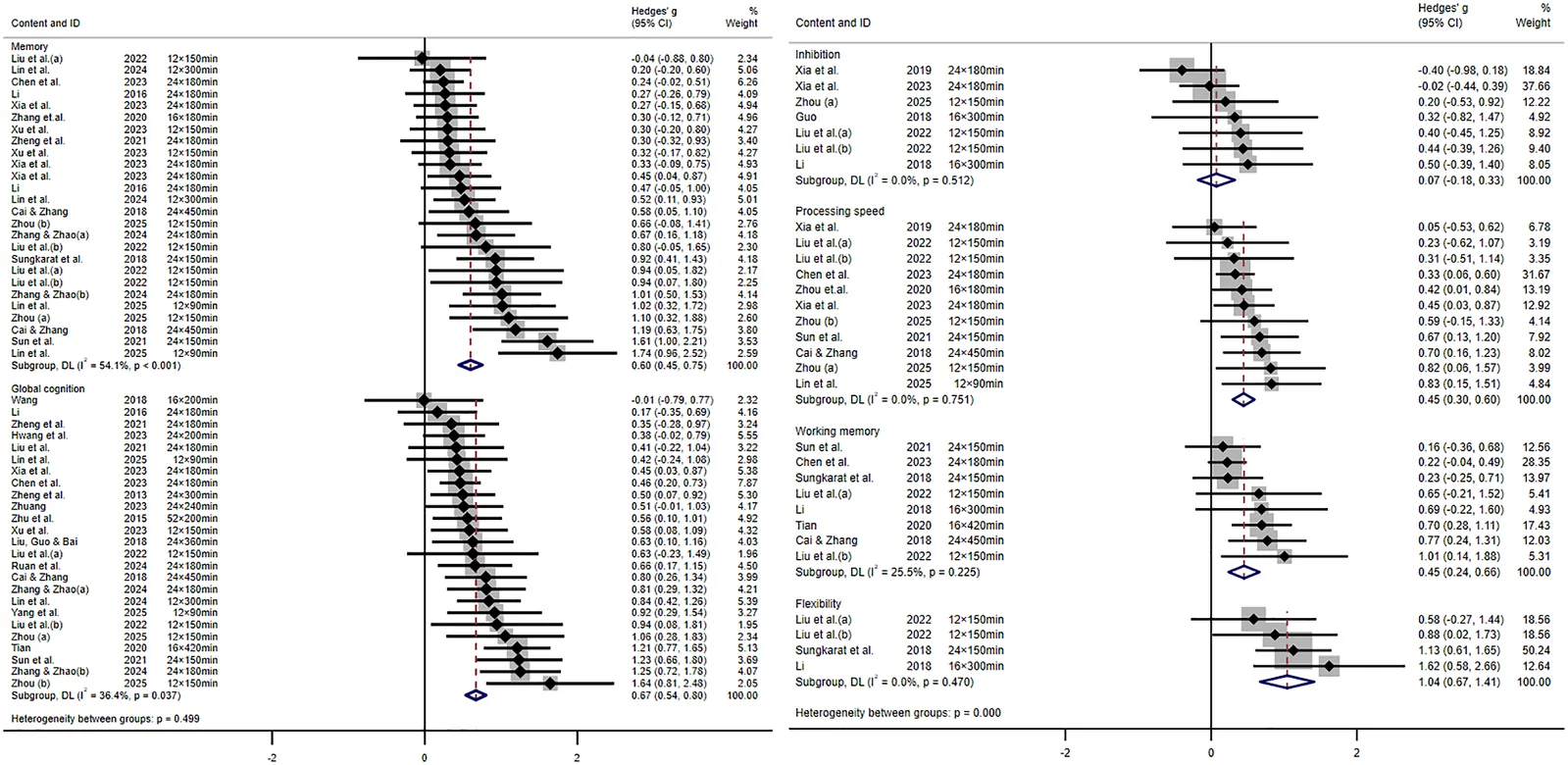
Forest plot of Mind-body exercise on cognition in older adult with MCI.

The pooled analysis revealed that mind-body exercise yielded domain-specific cognitive improvements in older individuals with MCI. The effect sizes were largest for cognitive flexibility (Hedges’ g = 1.04; 95% CI: [0.67, 1.41], I^2^ = 0%; n = 4 datasets from 3 RCTs), followed by moderate effect sizes in global cognition (g = 0.67; CI: [0.49, 0.78], I^2^ = 36.4%; n = 25 from 22 RCTs) and memory (g = 0.60; CI: [0.45, 0.75], I^2^ = 54.1%; n = 26 from 13 RCTs), and small yet significant effects for processing speed (g = 0.45; CI: [0.3, 0.6], I^2^ = 0%; n = 11 from 9 RCTs) and working memory (g = 0.45; CI: [0.24, 0.66], I^2^ = 25.5%; n = 8 from 7 RCTs). No significant effect was observed for inhibition (g = 0.07; CI: [−0.18, 0.33], p = 0.51, I^2^ = 0%; n = 7 from 6 RCTs) (Fig 3). Among the six cognitive domains, only memory exhibited substantial heterogeneity (I^2^ = 54.1%; p = 0.002). Subgroup analysis indicated that the heterogeneity was mainly driven by a single study [41] (S1 Fig in S1 File)*.* Notably, this study uniquely adopted the RBMT for the assessment of everyday behavioral memory [56] and used immersive virtual reality. Publication bias was detected for memory and working memory, warranting cautious interpretation of these domain-specific findings.

### Mind-body exercise dose-response on cognition

**Fig 4:**
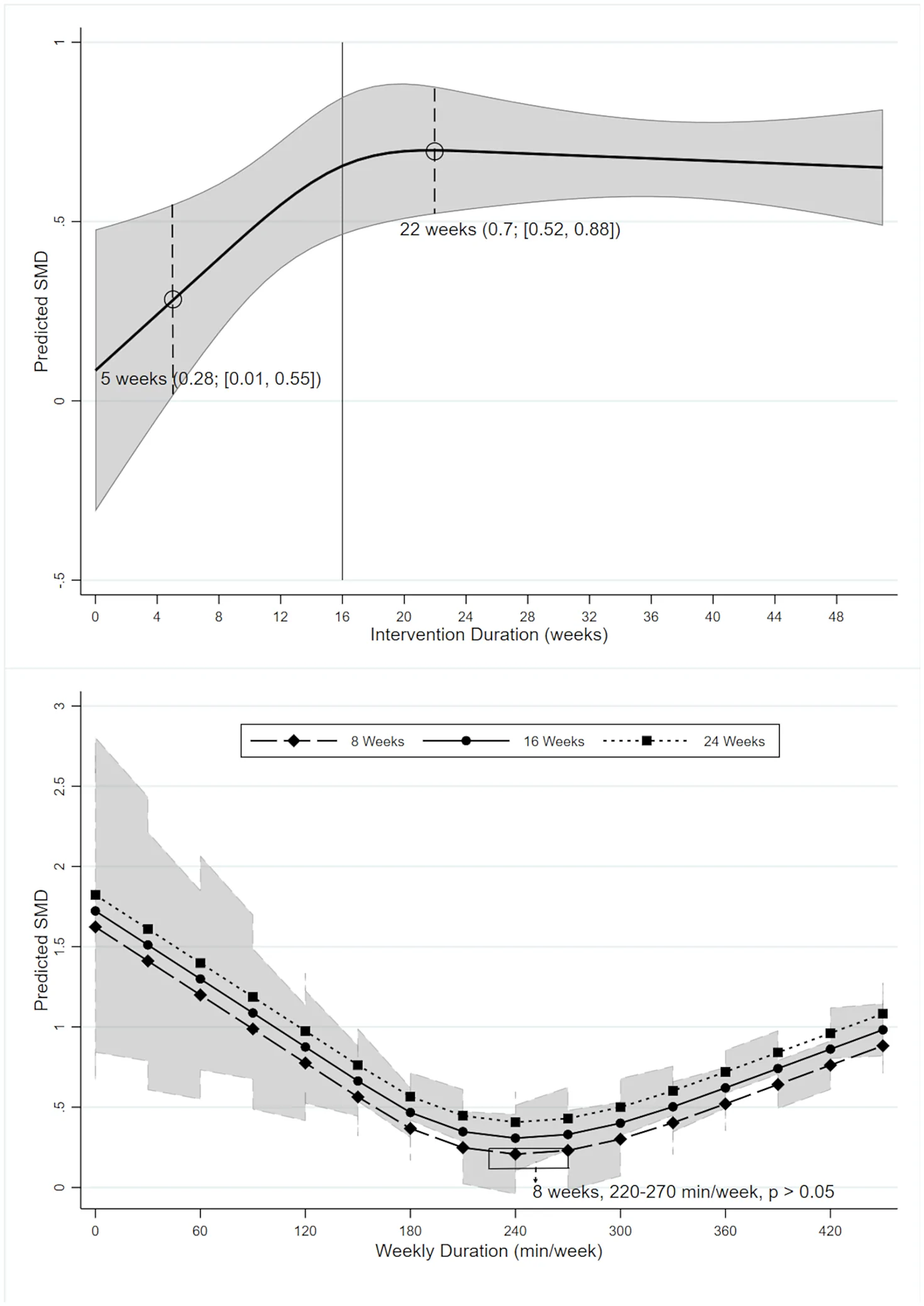
Mind-body exercise dose-response on global cognition. The upper panel depicts the week-response relationship; the lower panel depicts the weekly dosage-response relationship. Gray shaded areas indicate 95% CIs. Vertical dashed line in the upper panel mark the first significant effect, plateau onset, and peak effect. The shaded rectangle in the lower panel denotes the weekly dosage rang without significant effects over the 8-week protocol.

For global cognition, dose-response analysis demonstrated a significant ‘⦧’-shaped relationship for intervention length (Wald χ² (2) = 7.35, p < 0.05, knots (8, 16, 24), n = 38 data points). The effect reached statistical significance at week 5 (0.28; [0.01, 0.55]), increased at a diminishing rate after week 16, peaked at week 22 (0.7; [0.52, 0.88]), and then plateaued. This association became non-significance after adjustment for weekly or cumulative dose, suggesting confounding. In contrast, weekly dose exhibited a U-shaped relationship with cognition improvements (Wald χ² (2) = 22.98, p < 0.0001, knots (150 180 360)): effect sizes initially declined to a nadir at 240 min/week (0.37; [0.19, 0.55]) before increasing gradually. This nonlinear pattern remained significant after adjusting for intervention length (Wald χ^2^ (3) = 21.04, p < 0.0001) or cumulative dose (Wald χ^2^ (3) = 16.39, p < 0.0001), the model adjusting for intervention length had a lower QIC (12.8 vs 12.9), indicating superior fit. Notably, the U-shaped weekly dose-response relationship was positively modulated by program length (β = 0.012, p = 0.02) and cumulative dose (β = 4.9 × 10^-5^, p = 0.026;), reflecting an interaction. Exploratory analyses of the time-response pattern suggested that cognitive improvements firstly reached statistical significance at 5 weeks ignoring the weekly dose, but not at 8 weeks when weekly doses were 220–270 min/week (Fig 4). The earliest time point at which significant effects were detected for all weekly doses simultaneously was 11 weeks. No significant nonlinear association was found between cumulative dose and global cognition (p = 0.126, knots (1440 2880 4320 7200)).

**Fig 5:**
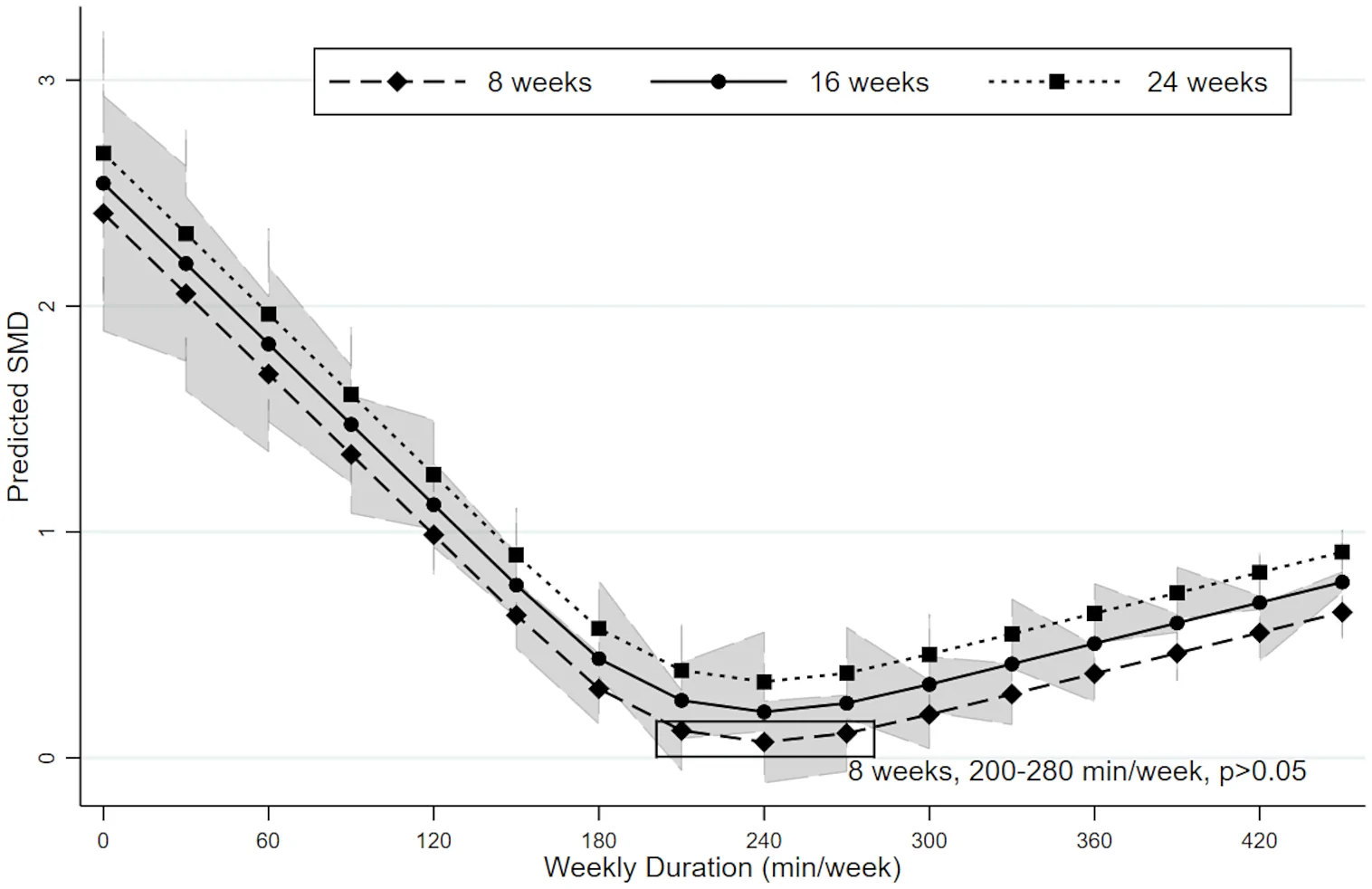
Mind-body exercise dose-response on memory. Gray shaded areas indicate 95% CIs. The shaded rectangle denotes the weekly dosage rang without significant effects over the 8-week protocol.

Memory exhibited a U-shaped dose-response pattern (Wald χ^2^ (2) = 52.8, p < 0.0001; knots (150, 180, 300), n = 37)*,* similar to that of global cognition, with the same nadir at 240 min/week. The relationship remained significant after adjusting for intervention length (Wald χ^2^ (3) = 61.58, p < 0.001; QIC: 11.36) or cumulative dose (Wald χ^2^ (3) = 69.02, p < 0.001; QIC: 11.43). Both intervention length (β = 0.015, p = 0.027) and cumulative dose (β = 5 × 10^-5^, p = 0.014) were positive associated with memory improvements, consistent with findings for global cognition. In time-point analyses of the dose-response relationship, no significant memory improvement was detected at 8 weeks for the weekly dose of 200–280 min/week, whereas significant improvement was observed across all weekly exercise doses at 15 weeks (Fig 5). No significant nonlinear relationship for intervention length was observed. In contrast, a significant nonlinear association was identified between cumulative dose with memory (Wald χ^2^ (2) = 12.56, p < 0.01; knots (1440 2880 4320)), although none of spline terms were statistically significant.

**Fig 6:**
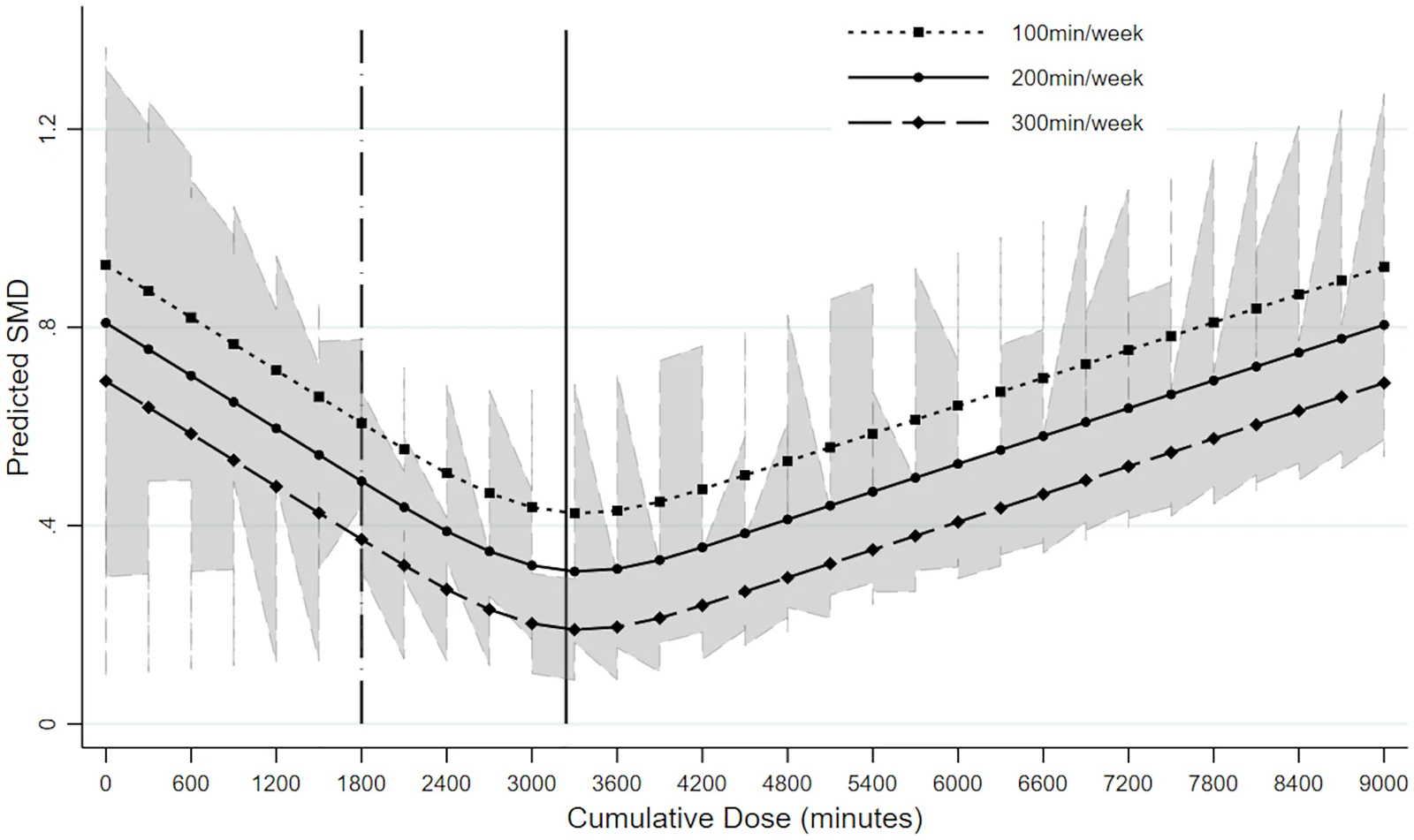
Mind-body exercise dose-response on processing speed. Gray shaded areas indicate 95% CIs. Vertically lines denote two knots: 1800 and 3240 minutes.

For processing speed, the data for intervention length and weekly dose were too clustered to permit automated knot generation, thus, dose-response analysis was limited to cumulative dose. The results revealed a significant U-shaped relationship between cumulative dose and processing speed improvements after adjusting for weekly dose (Wald χ^2^ (3) = 218.33, p < 0.001, knots (1800, 3240, 4320), n = 12). This U-shaped curve has a nadir at 3400 minutes, with the first spline term non-significant (p > 0.05), suggesting that nonlinearity was driven primarily by the latter segments. Weekly dose was independently negatively associated with processing speed gains (β = −0.0011, p = 0.026): each 100-minute increase in weekly dose corresponded to a 0.11-unit decrease in effect size (Fig 6).

Subgroup analyses revealed potential dose-dependent effects of cumulative dose on cognitive flexibility and working memory, albeit with differing statistical significance thresholds. For cognitive flexibility, a moderate and significant effect was observed at 1440–2880 minutes (g = 0.61; CI: [0.23, 0.99]), whereas working memory achieved a small but significant effect only when cumulative dose exceeded 2880 minutes (g = 0.26; CI: [0.07, 0.45]). Cognitive flexibility and working memory showed divergent dose-response patterns for intervention length and weekly dose, suggesting domain-specific mechanisms (Table 2)*.*

**Table 2:** Subgroup analysis of executive function outcomes.

	Working memory		Flexibility		Inhibition	
	SMD(95% CI)	N(datasets)	SMD(95% CI)	N(datasets)	SMD(95% CI)	N(datasets)
**All**	0.45(0.24, 0.66)	565(8)	1.04(0.67, 1.41)	136(4)	0.07(−0.18, 0.33)	262(7)
I^2^ (p)	25.5% (0.23)		0% (0.47)		0% (0.51)	
**weekly dose**						
≤ 150 min/wk	0.29 (0.04, 0.55)	228(5)	0.79(0.48, 1.1)	182(4)	0.33(−0.13, 0.79)	94(3)
151-300 min/wk	0.26 (0.00, 0.51)	238(2)	1.62(0.58, 2.66)	20(1)	−0.03(−0.36, 0.3)	168(4)
> 300 min/wk	0.58(0.35, 0.81)	308(4)				
**Length**						
≤ 8 wk	0.43 (0.02, 0.83)	96(1)	--	--	--	--
≈12 wk	0.63 (0.23, 1.03)	108(3)	0.73(0.12, 1.34)	50(2)	0.33(−0.13, 0.79)	94(3)
≈16 wk	0.48(0.09, 0.88)	182(3)	0.97(−0.08, 2.02)	86(2)	0.44(−0.27, 1.14)	32(2)
≈24 wk	0.3(0.07, 0.53)	399(4)	1.13(0.61, 1.65)	66(1)	0.11(−0.58, 0.36)	136(2)
> 24 wK	--	--	--	--	--	--
P _between groups_	0.549		0.267		0.147	
**total dose**						
≤1440 min	--	--	--	--	--	--
1441-2880 min	0.49(−0.03, 1.02)	116(3)	0.61(0.23, 0.99)	116(3)	0.33(−0.13, 0.79)	94(3)
2881-4320 min	0.26(0.07, 0.45)	437(4)	1.13(0.61, 1.65)	66(1)	−0.15(−0.5, 0.2)	136(2)
4321-5760 min	0.53(0.08, 0.99)	78(2)	1.62(0.58, 2.66)	20(1)	0.44(−0.27, 1.14)	32(2)
5761-7200 min	0.7(0.28,1.11)	96(1)	--	--	--	--
>7200 min	0.77(0.24, 1.31)	58(1)	--	--	--	--
P _between groups_	0.17		0.094		0.147	

### Sensitivity analysis

For memory, sensitivity analysis excluding the outlier study by Sun reduced heterogeneity in memory from 54.1% to 42.5% and attenuated the pooled effect size from 0.60 to 0.55, remained significant.

#### Publication bias.

**Fig 7:**
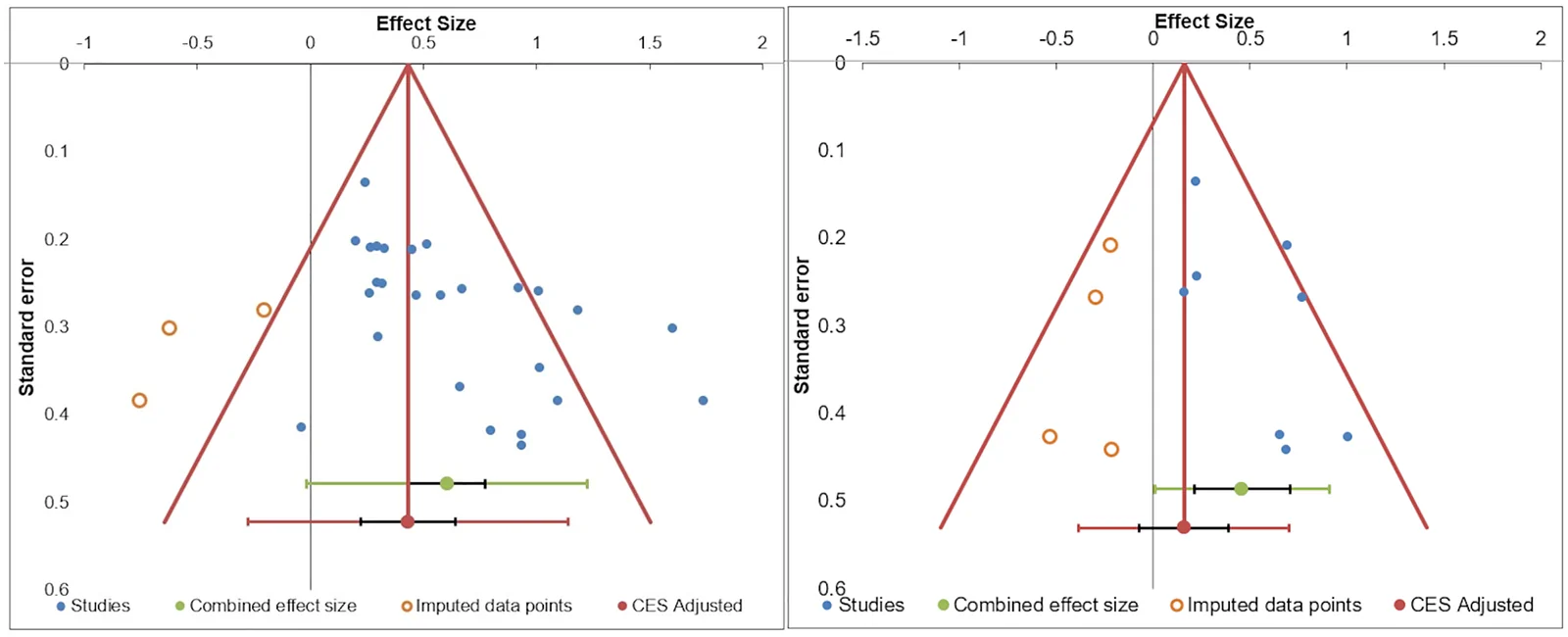
Funnel plot after trim-and-fill adjustment. The left panel is memory; the right panel is working memory.

Publication bias was examined across all six cognitive domains. Significant publication bias was detected for memory (Egger’s test, p = 0.023) and working memory, with evident funnel plot asymmetry observed in both domains. The trim-and-fill analysis based on a random-effects model was applied to correct for the identified publication bias. For memory, this procedure imputed three missing study, reducing the pooled effect size from 0.60 to 0.43 (95% CI: [0.22, 0.64]), while remaining statistically significant. Whereas, the adjusted 95% prediction interval (95% PI: [−0.28, 1.14]) crossed the null value, reflecting relatively large between-study variation in effect magnitudes. For working memory, four missing studies were imputed via the trim-and-fill method. The pooled effect size decreased from 0.45 to 0.16 (95% CI: [−0.10, 0.41]), resulting in loss of statistically significance (Fig 7).

#### GRADE assessment of the evidence.

A detailed Summary of GRADE assessments for all cognitive outcomes is presented in Table 3. Global cognition and processing speed were rated as moderate certainty (⊕⊕⊕⊖), each downgraded one level for serious risk of bias (lack of blinding and unclear allocation concealment). Working memory, inhibition and flexibility were rated as low certainty (⊕⊕⊖⊖). Working memory was downgraded for risk of bias and suspected publication bias, inhibition for risk of bias and serious imprecision; flexibility for risk of bias and serious imprecision (extremely small sample). Memory was rated as very low certainty (⊕⊖⊖⊖), downgraded three levels for serious risk of bias, serious inconsistency (I^2^ = 54.1%), and detected publication bias (Egger’s test p < 0.1)*.*

**Table 3:** Summary of GRADE evidence for cognitive outcomesoutcomes.

Outcome	RCTs(n)	Pts(n)	SMD[95% CI]	I²(%)	Certainty of evidence (GRADE)	Reasons for downgrading
Global cog	22	1574	0.67[0.49, 0.78]	36.4%	⊕⊕⊕⊖Moderate	Risk of bias (–1)
MEM	13	976	0.60[0.44, 0.75]	54.1%	⊕⊖⊖⊖ Very low	Risk of bias, inconsistency, publication bias (–3)
WK	7	530	0.45[0.24, 0.66]	25.5%	⊕⊕⊖⊖ Low	Risk of bias, publication bias (–2)
IC	6	234	0.07[–0.18, 0.33]	0%	⊕⊕⊖⊖ Low	Risk of bias, imprecision (–2)
CF	3	57	1.04[0.67, 1.41]	0%	⊕⊕⊖⊖ Low	Risk of bias, imprecision (–2)
PS	9	717	0.45[0.30, 0.60]	0%	⊕⊕⊕⊖Moderate	Risk of bias (–1)

## Discussion

This is the first meta-analysis to systematically quantify domain-specific dose-response relationships of mind-body exercise in older adults with MCI. Mind-body exercise produced strikingly domain-specific effects. Global cognition (0.67, [0.49 0.78]) showed a moderate improvement, and processing speed (0.45, [0.30 0.60]) a small-to-moderate improvement, both supported by moderate certainty evidence (GRADE ⊕⊕⊕⊖), downgraded only for risk of bias (unavoidable lack of blinding and adherence-related incomplete outcome data). Memory (0.60, [0.44 0.75]) exhibited a moderate effect but with very low certainty (⊕⊖⊖⊖), downgraded for risk of bias, inconsistency (I^2^ = 54.1%), and publication bias (Egger’s test p < 0.1). For executive function, mind-body exercise exerts heterogeneous therapeutic influences on three key subdomains. Cognitive flexibility benefited substantially (1.04, [0.67 1.41]), working memory showed small-to-moderate improvements (0.45, [0.24 0.66]), while inhibitory control exhibited negligible change (0.07, [−0.18 0.33]). The evidence supporting these results was rated low certainty (⊕⊕⊖⊖), due to the limited number of included trials.

Dose-response analyses identified a U-shaped association between mind-body exercise and cognitive gains in older adults with MCI, contrary to the inverted U curve reported by prior work [13,57,58]. Although, a ‘⦧’-shaped curve was initially detected between intervention duration and global cognitive gains, this relationship became statistically non-significant after adjusting for confounding influence of weekly dose. In contrast, global cognition exhibited a robust U-shaped weekly dose-response pattern, with a benefit nadir at approximately 240 min/week. Intervention duration positively modulated this relationship: each additional week of intervention added the overall effects size by ~0.011 SMD units. Statistical significance for cognitive improvement based on intervention duration was first achieved at 5 weeks of intervention. However, an 8-week regimen with weekly dosage of 220–270 min failed to reach significant cognitive benefits. The 240 min/week dose, which represents the curve’s trough, required ≥ 11 weeks to produce significant benefits. Even suboptimal weekly dose can achieve enhanced cognitive gain when paired with prolonged intervention duration.

The inconsistency between the curvilinear relationship in this study and previous works may stem from two methodological refinement: restriction to older adults with MCI and strict inclusion criteria. These approaches reduced heterogeneity and improved the certainty of evidence to moderate. Notably, the U-shaped dose-response relationship raises the hypothesis that mind-body exercise engages disparate biological pathways to regulate cognitive outcomes in older adults with MCI across varying dose range. Cognitive benefits linked to low weekly exercise dosage may be driven by hormesis [59]. By modulating neurotrophic factors and inflammatory signaling pathways, mind-body exercise enhances neuroplasticity and facilitates cognitive improvements [60–62]. Highly weekly doses may enhance cognition by optimizing resting-state neural oscillations and task-related neural efficiency [63]. Long-term intervention drives cumulative structural modifications in pivotal brain regions such as hippocampus and anterior cingulate cortex [39,46,64,65], enhances functional connectivity between associated brain areas [64], and builds up sustained neuroprotective effects at the molecular level [65]. These cascading biological effects why prolonged interventions can elicit substantial cognitive improvements even under suboptimal moderate weekly exercise doses.

Furthermore, the similar dose-response emerged for memory, albeit supported by evidence of very low certainty. For processing speed (moderate certainty), a U-shaped cumulative dose-response relationship was identified, with a nadir at 3400 total minutes. Weekly dose negatively moderated this relationship: each 100 min/week increment was associated with an ~ 0.11-unit reduction in effect size. Notably, available weekly dose data were largely confined to 150 and 180 min/week, which lies on the descending limb of the U-shaped weekly dose curve seen for global cognition. Thus, the apparent negative moderation may reflect the left-hand portion of potential full U-shape rather than a genuinely inverse effect. This remains an open question requiring studies with wider variation in weekly dose.

## Limitations

This study also has several limitations. First, the number of studies and participants was small for low- and very-low-certainty domains (memory, working memory, inhibition, flexibility), and the high risk of bias in these studies precludes firm conclusions. Second, the unified weekly dose metric (min/week) facilitates cross-protocol comparison but cannot separate the independent contributions of exercise frequency versus duration per session. Finally, the mechanistic basis for the observed interactions among intervention parameters remains unknown and requires further investigation.

## Clinical implications and future research

The present dose-response analysis provides novel, clinically meaningful evidence for mind-body exercise intervention in older adults with MCI. This study with the RCS analysis identified a robust U-shaped relationship between weekly exercise dose and global cognition, showing cognitive benefits that cognitive gains reach a nadir at a weekly dose close to 240 min. Notably, intervention duration positively modulates this dose-response relationship: extending the total intervention duration to 11 weeks can offset the lower efficacy associated with the 240-min/week dose. These findings, however, reflect study-level exploratory associations, not individual-level causal effects; prospective validation is needed before clinical application.

However, the similar dose-response association observed for memory rests upon evidence with very low certainty. For processing speed, available weekly exercise doses clustered below 240 min/week, with insufficient high-dose data. Further investigations are warranted to verify the dose-response relationships between mind-body exercise and other cognitive subdomains in older adults with MCI, and to elucidate the neurobiological mechanisms, providing evidence for tailor exercise intervention prescription.

## Supporting information

S1 File Supplementary materials for this study. This supporting information file contains multiple supplementary items, including subgroup comparison analyses, sensitivity tests, complete literature search strategies, raw extracted datasets, and annotated analysis code. Supplementary materials associated with the manuscript, including: 1) subgroup analyses examining effects estimates; 2).
(DOCX)
