# Correction: Nonlinear dose-response of mind-body exercise in older adults with mild cognitive impairment: A systematic review and meta-analysis

**DOI:** 10.1371/journal.pone.0361388

**Published:** 2026-10-22

**Authors:** Yan-Fang Peng, Xi Chen, Guo-Qing Zhu, Guang-Cai Qu

In the Discussion section, the last two references in the sixth sentence of the third paragraph should have cited references 66 and 67 instead of 64 and 65. The correct sentence is: Long-term intervention drives cumulative structural modifications in pivotal brain regions such as hippocampus and anterior cingulate cortex [39,46,64,65], enhances functional connectivity between associated brain areas [66], and builds up sustained neuroprotective effects at the molecular level [67].

66. Liu J, Tao J, Liu W, Huang J, Xue X, Li M, et al. Different modulation effects of Tai Chi Chuan and Baduanjin on resting-state functional connectivity of the default mode network in older adults. Soc Cogn Affect Neurosci. 2019;14: 217–224. doi:10.1093/scan/nsz001

67. Gan J, He J, Zhou K, Shang Z, Dong G, Bao D, et al. Effects of traditional Chinese exercises on brain-derived neurotrophic factor in middle-aged and older adults: A systematic review and meta-analysis of randomized controlled trials. J Exerc Sci Fit. 2025;23: 32–41. doi:10.1016/j.jesf.2024.12.004
