# Supplementary material for: Nonlinear dose-response of mind-body exercise in older adults with mild cognitive impairment: A systematic review and meta-analysis
Source: PLoS One. 2026 Sep 25;21(9):e0350867. doi: 10.1371/journal.pone.0350867 (PMC13614650; doi:10.1371/journal.pone.0350867)
Supplement: Supplementary file 1 [file pone.0350867.s001.docx]

S1 Figure: Forest plot of mind-body exercise on memory based on content

A	B

C	D

S2 Figure: The funnel plot of cognition

(A: global cognition; B: processing speed; C: cognitive flexibility; D: inhibition control)

S3 Figure. Subgroup analysis of working memory after adjustment

S1 Table: Search Strategy

| Content | Results |
| --- | --- |
| Data sources | English databases: Pubmed, Embase, Scopus Medline, Web of Science, and Cochrane Library |
|  | Chinese databases: China National Knowledge Infrastructure (CNKI), Wanfang Data Knowledge Service Platform (Wanfang), and Chongqing VIP (VIP) |
| Strategy | (cognitive impairment OR mild cognitive impairment OR MCI OR mild cognitive disorder OR mild cognitive decline OR mild cognitive dysfunction OR mild cognitive disorder OR MCD) AND (martial art OR Wushu OR Wu shu OR Tai Chi OR Taichi OR Tai Chi Chuan OR Taijiquan OR Tai ji OR Taichiquan OR Baduanjin OR Liuzijue OR Six healing sounds OR Yijinjing OR Wuqinxi OR Qigong OR fitness qigong OR Zhanzhuang OR Mind-body exercise OR Mind-body therapies OR Mind-body OR traditional Chinese exercise OR traditional Chinese physical exercise OR traditional Chinese medical exercise) |
| Time span | From inception to September 15, 2025 |
| Language | English or Chinese |
| Results | 2401 |

S2 Table: Comparative analyses of weighted versus unweighted models

|  | Model | Wald x2  (unweight） | Wald x2  (1/SE^2^ weight） | Consistent  (yes/no) |
| --- | --- | --- | --- | --- |
| global cognition | week | 7.35 | 12.79 | yes |
|  | weekly dose* week | 21.04 | 34.91 | yes |
|  | Weekly dose* total dose | 16.39 | 24.19 | yes |
|  | Total dose | 5.72# | 14.17 | no |
|  |  |  |  |  |
| memory | week | 3.23 # | 3.48 # | yes |
|  | weekly dose* week | 61.58 | 50.24 | yes |
|  | weekly dose* total dose | 69.02 | 64.04 | yes |
|  | total dose | 12.56 | 4.08 # | no |
|  |  |  |  |  |
| processing speed | total dose* weekly dose | 218.33 | 123.91 | yes |

#: no significant

Code Appendix

gen intensity_week = freq * dur

gen total_dose = intensity_week * week

gen dose_infor = string(week) + "×" + string(intensity_week) + "min"

gen pooled_sd = sqrt( ((n_int - 1) * sd_int^2 + (n_con - 1) * sd_con^2) / (n_int + n_con - 2) )

gen smd = (change_int - change_con) / pooled_sd

gen se_smd = sqrt( (n_int + n_con)/(n_int * n_con) + smd^2/(2*(n_int + n_con)) )

Forest:

metan n_int change_int sd_int n_con change_con sd_con, lcols(id year dose_infor) by(content) random hedges nooverall effect("Hedges' g")

dose-response analysis:

mkspline s_intensity_week = intensity_week, cubic nknots(n) displayknots

xtgee smd s_intensity_week* week, family(gaussian) link(identity) vce(robust) i(number)

mkspline rcs_intensity = intensity_week, knots(*knot1 knot2 knot3* ) cubic displayknots

xtgee smd rcs_intensity1 rcs_intensity2 week, family(gaussian) link(identity) corr(exchangeable) vce(robust) i(number)

clear

set obs 16

gen intensity_week = 0 + (_n-1)*30

expand 3

bysort intensity_week: gen week = 8 + 8*(_n-1)

mkspline rcs_intensity = intensity_week, knots(*knot1 knot2 knot3*) cubic

predict smd_pred

predict yhat

predict stdp, stdp

gen lb = yhat - 1.96 * stdp

gen ub = yhat + 1.96 * stdp

sort week intensity_week

twoway (rarea lb ub intensity_week, sort fcolor(gs14%40) lcolor(gs14) lwidth(vthin)) || (connected smd_pred intensity_week if week == 8, sort lcolor(orange) mcolor(orange) msymbol(O)) (connected smd_pred intensity_week if week == 16, lcolor(maroon) mcolor(maroon) msymbol(O)) (connected smd_pred intensity_week if week == 24, sort lcolor(green) mcolor(green) msymbol(O)), xtitle("Weekly Duration (min/week)”) ytitle("Predicted SMD") legend(order(2 "Week 8" 3 "Week 16" 4 "Week 24"))

1/SE^2^ weight

gen inv_wt_obs = 1/(se_smd^2)

bysort number: egen inv_wt = mean (inv_wt_obs)

mkspline rcs_intensity = intensity_week, knots(knot1 knot2 knot2) cubic displayknots

xtgee smd rcs_intensity1 rcs_intensity2 week [pweight=inv_wt], family(gaussian) link(identity) corr(exchangeable) vce(robust) i(number)

Data Appendix

| **Global cognition** | | | | | | | | | | | | | |
| --- | --- | --- | --- | --- | --- | --- | --- | --- | --- | --- | --- | --- | --- |
| No. | Author | year | intervention | | | | Experimental group | | | Control group | | | content |
|  |  |  | type | freq | dur | week | n_ | Mean-c | Sd-c | n_ | Mean-c | Sd-c |  |
| 6 | Cai & Zhang | 2018 | The others | 5 | 90 | 24 | 28 | 1.8 | 2.29 | 30 | 0 | 2.15 | Global cognition |
| 43 | Chen et al. | 2023 | TaiChi | 3 | 60 | 24 | 107 | 2.61 | 2.95 | 111 | 1.2 | 3.09 | Global cognition |
| 4 | Hwang et al. | 2023 | TaiChi | 4 | 50 | 24 | 41 | 1.8 | 5.9 | 56 | 0.2 | 2.1 | Global cognition |
| 10 | Li | 2016 | Baduanjin | 3 | 60 | 24 | 28 | 2.32 | 2.87 | 29 | 1.82 | 3.07 | Global cognition |
| 32 | Lin et al. | 2024 | TaiChi | 5 | 60 | 12 | 48 | 3.54 | 2.72 | 48 | 1.4 | 2.32 | Global cognition |
| 2 | Liu et al.(a) | 2022 | TaiChi | 3 | 50 | 12 | 17 | 1.6 | 3.43 | 8 | -0.5 | 2.69 | Global cognition |
| 5 | Liu et al.(b) | 2022 | TaiChi | 3 | 50 | 12 | 16 | 1.9 | 2.33 | 9 | -0.5 | 2.68 | Global cognition |
| 19 | Liu et al. | 2021 | Baduanjin | 3 | 60 | 24 | 20 | 2.1 | 2.21 | 20 | 1.2 | 2.07 | Global cognition |
| 12 | Liu, Guo & Bai | 2018 | Baduanjin | 6 | 60 | 24 | 28 | 3.18 | 1.75 | 29 | 1.81 | 2.47 | Global cognition |
| 18 | Sun et al. | 2021 | Baduanjin | 3 | 50 | 24 | 29 | 4.38 | 2.86 | 28 | 0.76 | 2.93 | Global cognition |
| 20 | Tian | 2020 | TaiChi | 7 | 60 | 16 | 46 | 1.69 | 1.23 | 50 | 0.34 | 0.98 | Global cognition |
| 31 | Wang | 2018 | Baduanjin | 5 | 40 | 16 | 15 | -0.01 | 1.78 | 11 | 0.01 | 1.44 | Global cognition |
| 42 | Xia et al. | 2023 | Baduanjin | 3 | 60 | 24 | 45 | 3.94 | 7.93 | 45 | -2.02 | 16.64 | Global cognition |
| 3 | Xu et al. | 2023 | Baduanjin | 3 | 50 | 12 | 31 | 2 | 2.74 | 32 | 0.25 | 3.16 | Global cognition |
| 35 | Zhang & Zhao(a) | 2024 | Baduanjin | 3 | 60 | 24 | 48 | 3.88 | 3.66 | 23 | 0.84 | 3.85 | Global cognition |
| 36 | Zhang & Zhao(b) | 2024 | Baduanjin | 3 | 60 | 24 | 50 | 5.46 | 3.55 | 24 | 0.84 | 3.85 | Global cognition |
| 1 | Zheng et al. | 2013 | The others | 5 | 60 | 24 | 45 | 2.42 | 5.11 | 43 | -0.03 | 4.63 | Global cognition |
| 15 | Zheng et al. | 2021 | Baduanjin | 3 | 60 | 24 | 20 | 2 | 2.16 | 20 | 0.97 | 3.48 | Global cognition |
| 40 | Zhu et al. | 2015 | Baduanjin | 5 | 40 | 52 | 37 | 3.81 | 5.48 | 41 | 0.92 | 4.81 | Global cognition |
| 22 | Zhuang | 2023 | Baduanjin | 7 | 34.2857 | 24 | 29 | 1.17 | 3.24 | 30 | -0.5 | 3.22 | Global cognition |
| 47 | Yang et al. | 2025 | Baduanjin | 3 | 30 | 12 | 20 | 2.3 | 2.48861 | 25 | 0.08 | 2.29632 | Global cognition |
| 49 | Zhou (a) | 2025 | TaiChi | 3 | 50 | 12 | 22 | 2.6 | 3.17013 | 11 | -0.7 | 2.78345 | Global cognition |
| 50 | Zhou (b) | 2025 | TaiChi | 3 | 50 | 12 | 22 | 4.9 | 3.55161 | 11 | -0.7 | 2.78345 | Global cognition |
| 52 | Ruan et al. | 2024 | TaiChi | 3 | 60 | 24 | 33 | 2.51 | 2.44845 | 35 | 0.8 | 2.67513 | Global cognition |
| 53 | Lin et al. | 2025 | TaiChi | 3 | 30 | 12 | 18 | 2.28 | 1.41 | 18 | 1.78 | 0.83 | Global cognition |
| 6 | Cai & Zhang | 2018 | The others | 5 | 90 | 12 | 28 | 1.5 | 2.07 | 30 | -0.5 | 2 | Global cognition |
| 20 | Tian | 2020 | TaiChi | 7 | 60 | 8 | 46 | 0.91 | 0.94 | 50 | 0.38 | 1.05 | Global cognition |
| 35 | Zhang & Zhao(a) | 2024 | Baduanjin | 3 | 60 | 8 | 48 | 1.06 | 3.77 | 23 | 0.83 | 3.6 | Global cognition |
| 35 | Zhang & Zhao(a) | 2024 | Baduanjin | 3 | 60 | 16 | 48 | 2.3 | 3.86 | 23 | 0.78 | 3.85 | Global cognition |
| 36 | Zhang & Zhao(b) | 2024 | Baduanjin | 3 | 60 | 8 | 50 | 1.23 | 3.84 | 24 | 0.83 | 3.6 | Global cognition |
| 36 | Zhang & Zhao(b) | 2024 | Baduanjin | 3 | 60 | 16 | 50 | 3.02 | 3.7 | 24 | 0.78 | 3.85 | Global cognition |
| 40 | Zhu et al. | 2015 | Baduanjin | 5 | 40 | 4 | 37 | 0.93 | 6.09 | 41 | 0.12 | 5.6 | Global cognition |
| 40 | Zhu et al. | 2015 | Baduanjin | 5 | 40 | 12 | 37 | 2.12 | 6.4 | 41 | 0.77 | 5.36 | Global cognition |
| 40 | Zhu et al. | 2015 | Baduanjin | 5 | 40 | 24 | 37 | 3.24 | 5.91 | 41 | 0.55 | 4.9 | Global cognition |
| 40 | Zhu et al. | 2015 | Baduanjin | 5 | 40 | 39 | 37 | 3.56 | 5.69 | 41 | 0.81 | 5.69 | Global cognition |
| 22 | Zhuang | 2023 | Baduanjin | 7 | 34.2857 | 12 | 29 | 0.17 | 3.24 | 30 | -0.42 | 3.13 | Global cognition |
| 10 | Li | 2016 | Baduanjin | 3 | 60 | 8 | 28 | 0.84 | 2.81 | 29 | 1.21 | 3.21 | Global cognition |
| **10** | Li | 2016 | Baduanjin | 3 | 60 | 16 | 28 | 1.71 | 2.79 | 29 | 2.63 | 2.62 | Global cognition |
| **memory** | | | | | | | | | | | | | |
| No. | Author | year | intervention | | | | Experimental group | | | Control group | | | content |
|  |  |  | type | freq | dur | week | n_ | Mean-c | Sd-c | n_ | Mean-c | Sd-c |  |
| 6 | Cai & Zhang | 2018 | The others | 5 | 90 | 24 | 28 | 1.8 | 2.29 | 30 | 0.5 | 2.15 | Immediate recall |
| 10 | Li | 2016 | Baduanjin | 3 | 60 | 24 | 28 | 0.61 | 3.09 | 29 | -0.27 | 3.44 | Immediate recall |
| 32 | Lin et al. | 2024 | TaiChi | 5 | 60 | 12 | 48 | 0.31 | 2.69 | 48 | -0.23 | 2.6 | Immediate recall |
| 2 | Liu et al.(a) | 2022 | TaiChi | 3 | 50 | 12 | 17 | -0.8 | 6 | 8 | -0.6 | 3.73 | Immediate recall |
| 4 | Liu et al.(b) | 2022 | TaiChi | 3 | 50 | 12 | 16 | 2.6 | 3.05 | 9 | -0.6 | 3.73 | Immediate recall |
| 42 | Xia et al. | 2023 | Baduanjin | 3 | 60 | 24 | 45 | 8.74 | 8.64 | 45 | 6.06 | 11.03 | Immediate recall |
| 3 | Xu et al. | 2023 | Baduanjin | 3 | 50 | 12 | 31 | 0.67 | 2.57 | 32 | 0 | 1.81 | Immediate recall |
| 35 | Zhang & Zhao(a) | 2024 | Baduanjin | 3 | 60 | 24 | 48 | 1.5 | 1.52 | 23 | 0.55 | 1.09 | Immediate recall |
| 36 | Zhang & Zhao(b) | 2024 | Baduanjin | 3 | 60 | 24 | 50 | 1.77 | 1.24 | 24 | 0.55 | 1.09 | Immediate recall |
| 53 | Lin et al. | 2025 | TaiChi | 3 | 30 | 12 | 18 | 2.06 | 2.55 | 18 | -0.28 | 1.9 | Immediate recall |
| 10 | Li | 2016 | Baduanjin | 3 | 60 | 8 | 28 | -0.04 | 3.3 | 29 | -0.35 | 3.36 | Immediate recall |
| 35 | Zhang & Zhao(a) | 2024 | Baduanjin | 3 | 60 | 8 | 48 | 0.69 | 1.24 | 23 | 0.12 | 1.15 | Immediate recall |
| 36 | Zhang & Zhao(b) | 2024 | Baduanjin | 3 | 60 | 8 | 50 | 0.77 | 1.18 | 24 | 0.12 | 1.15 | Immediate recall |
| 10 | Li | 2016 | Baduanjin | 3 | 60 | 16 | 28 | 1.13 | 3.28 | 29 | -0.08 | 3.5 | Immediate recall |
| 35 | Zhang & Zhao(a) | 2024 | Baduanjin | 3 | 60 | 16 | 48 | 1 | 1.18 | 23 | 0.31 | 1.07 | Immediate recall |
| 36 | Zhang & Zhao(b) | 2024 | Baduanjin | 3 | 60 | 16 | 50 | 1.28 | 1.19 | 24 | 0.31 | 1.07 | Immediate recall |
| 6 | Cai & Zhang | 2018 | The others | 5 | 90 | 12 | 28 | 1.5 | 2.07 | 30 | 0 | 2 | Immediate recall |
| 6 | Cai & Zhang | 2018 | The others | 5 | 90 | 24 | 28 | 5.6 | 4.44 | 30 | 0.3 | 4.36 | Delay recall |
| 10 | Li | 2016 | Baduanjin | 3 | 60 | 24 | 28 | 3.32 | 3.54 | 29 | 1.67 | 3.33 | Delay recall |
| 2 | Liu et al.(a) | 2022 | TaiChi | 3 | 50 | 12 | 17 | 2.1 | 3.18 | 8 | -0.7 | 2.1 | Delay recall |
| 4 | Liu et al.(b) | 2022 | TaiChi | 3 | 50 | 12 | 16 | 1.2 | 2.39 | 9 | -0.7 | 2.1 | Delay recall |
| 39 | Sungkarat et al. | 2018 | TaiChi | 3 | 50 | 24 | 33 | 0.57 | 0.46 | 33 | 0.14 | 0.46 | Delay recall |
| 42 | Xia et al. | 2023 | Baduanjin | 3 | 60 | 24 | 45 | 3.54 | 3.51 | 45 | 0.53 | 8.6 | Delay recall |
| 3 | Xu et al. | 2023 | Baduanjin | 3 | 50 | 12 | 31 | 1 | 4.03 | 32 | 0 | 1.66 | Delay recall |
| 44 | Zhang et.al. | 2020 | TaiChi | 4 | 45 | 16 | 43 | 1.33 | 1.98 | 49 | 0.83 | 1.35 | Delay recall |
| 49 | Zhou (a) | 2025 | TaiChi | 3 | 50 | 12 | 22 | 1.05 | 1.30931 | 11 | -0.31 | 0.957202 | Delay recall |
| 50 | Zhou (b) | 2025 | TaiChi | 3 | 50 | 12 | 22 | 1.26 | 1.38568 | 11 | -0.31 | 3.54065 | Delay recall |
| 53 | Lin et al. | 2025 | TaiChi | 3 | 30 | 12 | 18 | 2.44 | 1.62 | 18 | -0.33 | 1.49 | Delay recall |
| 10 | Li | 2016 | Baduanjin | 3 | 60 | 8 | 28 | 1.88 | 3.14 | 29 | 0.7 | 2.95 | Delay recall |
| 10 | Li | 2016 | Baduanjin | 3 | 60 | 16 | 28 | 3.14 | 3.36 | 29 | 1.95 | 3.37 | Delay recall |
| 14 | Sungkarat et al. | 2016 | TaiChi | 3 | 50 | 15 | 33 | 10.1 | 8.62 | 33 | 6.5 | 11.49 | Delay recall |
| 6 | Cai & Zhang | 2018 | The others | 5 | 90 | 12 | 28 | 3.4 | 4.56 | 30 | 0.4 | 4.42 | Delay recall |
| 32 | Lin et al. | 2024 | TaiChi | 5 | 60 | 12 | 48 | 2.67 | 14.38 | 48 | -3.5 | 15.21 | WMS-MQ |
| 42 | Xia et al. | 2023 | Baduanjin | 3 | 60 | 24 | 45 | 13.3 | 12.42 | 45 | 8.66 | 15.23 | WMS-MQ |
| 15 | Zheng et al. | 2021 | Baduanjin | 3 | 60 | 24 | 20 | 14.69 | 14.83 | 20 | 9.63 | 17.83 | WMS-MQ |
| 43 | Chen et al | 2023 | TaiChi | 3 | 60 | 24 | 107 | 8.81 | 12.38 | 111 | 5.38 | 15.33 | WMS-MQ |
| 18 | Sun et al. | 2021 | Baduanjin | 3 | 50 | 24 | 29 | 4.22 | 2.16 | 28 | 0.96 | 1.82 | RBMT |
| **Inhibition** | | | | | | | | | | | | | |
| No. | Author | year | intervention | | | | Experimental group | | | Control group | | | content |
|  |  |  | type | freq | dur | week | n_ | Mean-c | Sd-c | n_ | Mean-c | Sd-c |  |
| 41 | Guo | 2018 |  | 5 | 60 | 16 | 6 | 7.01 | 27.04 | 6 | -2.44 | 26.68 | Inhibition |
| 30 | Li | 2018 |  | 5 | 60 | 16 | 10 | 19.14 | 19.8 | 10 | 7.3 | 24.86 | Inhibition |
| 2 | Liu et al.(a) | 2022 |  | 3 | 50 | 12 | 17 | 11.6 | 46.37 | 8 | -8.1 | 48.87 | Inhibition |
| 3 | Liu et al.(b) | 2022 |  | 3 | 50 | 12 | 16 | 11.1 | 38.66 | 9 | -8.1 | 48.87 | Inhibition |
| 23 | Xia et al. | 2019 |  | 3 | 60 | 24 | 23 | -31.34 | 99.23 | 23 | 44.83 | 245.5 | Inhibition |
| 42 | Xia et al. | 2023 |  | 3 | 60 | 24 | 45 | 4.08 | 179.324 | 45 | 10.76 | 347.398 | Inhibition |
| 49 | Zhou (a) | 2025 |  | 3 | 50 | 12 | 22 | 2.56 | 129.267 | 11 | -24.57 | 146.477 | Inhibition |
| 50 | Zhou (b) | 2025 |  | 3 | 50 | 12 | 22 | 538.49 | 139.528 | 11 | -24.57 | 146.477 | Inhibition |
| **Processing speed** | | | | | | | | | | | | | |
| No. | Author | year | intervention | | | | Experimental group | | | Control group | | | content |
|  |  |  | type | freq | dur | week | n_ | Mean-c | Sd-c | n_ | Mean-c | Sd-c |  |
| 6 | Cai & Zhang | 2018 |  | 5 | 90 | 24 | 28 | 12.5 | 11.81 | 30 | 0.5 | 20.74 | Processing speed |
| 43 | Chen et al. | 2023 |  | 3 | 60 | 24 | 107 | 4.61 | 10.38 | 111 | 1.31 | 9.468 | Processing speed |
| 2 | Liu et al.(a) | 2022 |  | 3 | 50 | 12 | 17 | 9.8 | 37.29 | 8 | 1.6 | 29.7 | Processing speed |
| 3 | Liu et al.(b) | 2022 |  | 3 | 50 | 12 | 16 | 11.2 | 29.5 | 9 | 1.6 | 29.7 | Processing speed |
| 18 | Sun et al. | 2021 |  | 3 | 50 | 24 | 29 | 8.35 | 9.72 | 28 | 1.89 | 9.38 | Processing speed |
| 42 | Xia et al. | 2023 |  | 3 | 60 | 24 | 45 | 7 | 16.26 | 45 | -1.44 | 20.49 | Processing speed |
| 44 | Zhou et.al. | 2020 |  | 4 | 45 | 16 | 43 | 9.67 | 21.68 | 49 | 0.33 | 22.16 | Processing speed |
| 23 | Xia et al. | 2019 |  | 3 | 60 | 24 | 23 | 4.5 | 5.85 | 23 | 4.13 | 9.69 | Processing speed |
| 53 | Lin et al. | 2025 |  | 3 | 30 | 12 | 18 | 20 | 8.15 | 18 | -0.5 | 33.15 | Processing speed |
| 49 | Zhou (a) | 2025 |  | 3 | 50 | 12 | 22 | 0.3 | 0.51 | 11 | -0.21 | 0.65 | Processing speed |
| 50 | Zhou (b) | 2025 |  | 3 | 50 | 12 | 22 | 0.16 | 0.51 | 11 | -0.21 | 0.65 | Processing speed |
| 6 | Cai & Zhang | 2018 |  | 5 | 90 | 12 | 28 | 4.7 | 16.1 | 30 | 1.1 | 19.78 | processing speed |
| **working memory** | | | | | | | | | | | | | |
| No. | Author | year | intervention | | | | Experimental group | | | Control group | | | content |
|  |  |  | type | freq | dur | week | n_ | Mean-c | Sd-c | n_ | Mean-c | Sd-c |  |
| 6 | Cai & Zhang | 2018 |  | 5 | 90 | 24 | 28 | 20.2 | 30.27 | 30 | -1 | 23.75 | working memory |
| 43 | Chen et al. | 2023 |  | 3 | 60 | 24 | 107 | 22.16 | 86.01 | 111 | 3.26 | 83.71 | working memory |
| 30 | Li | 2018 |  | 5 | 60 | 16 | 10 | 169.24 | 162.12 | 10 | 37.16 | 201.34 | working memory |
| 2 | Liu et al.(a) | 2022 |  | 3 | 50 | 12 | 17 | 52.4 | 74.54 | 8 | 5.7 | 54.46 | working memory |
| 3 | Liu et al.(b) | 2022 |  | 3 | 50 | 12 | 16 | 60.2 | 51.18 | 9 | 5.7 | 54.46 | working memory |
| 18 | Sun et al. | 2021 |  | 3 | 50 | 24 | 29 | 10.37 | 53.08 | 28 | 1.78 | 51.828 | working memory |
| 39 | Sungkarat et al. | 2018 |  | 3 | 50 | 24 | 33 | 0.07 | 0.172 | 33 | 0.03 | 0.172 | working memory |
| 20 | Tian | 2020 |  | 7 | 60 | 16 | 46 | 13.14 | 20.18 | 50 | -2.06 | 22.97 | working memory |
| 14 | Sungkarat et al. | 2016 |  | 3 | 50 | 15 | 33 | 1.4 | 3.45 | 33 | 0.9 | 3.45 | working memory |
| 6 | Cai & Zhang | 2018 |  | 5 | 90 | 12 | 28 | 16.5 | 27.99 | 30 | 2.5 | 29.34 | working memory |
| 20 | Tian | 2020 |  | 7 | 60 | 8 | 46 | 9.6 | 20.17 | 50 | 0.41 | 22.43 | working memory |
| **flexibility** | | | | | | | | | | | | | |
| No. | Author | year | intervention | | | | Experimental group | | | Control group | | | content |
|  |  |  | type | freq | dur | week | n_ | Mean-c | Sd-c | n_ | Mean-c | Sd-c |  |
| 30 | Li | 2018 |  | 5 | 60 | 16 | 10 | 93.25 | 73.99 | 10 | -95.27 | 139.22 | flexibility |
| 2 | Liu et al.(a) | 2022 |  | 3 | 50 | 12 | 17 | 42.4 | 67.96 | 8 | 5.1 | 44.59 | flexibility |
| 3 | Liu et al.(b) | 2022 |  | 3 | 50 | 12 | 16 | 47.9 | 48.58 | 9 | 5.1 | 44.59 | flexibility |
| 39 | Sungkarat et al. | 2018 |  | 3 | 50 | 24 | 33 | 0.4 | 0.35 | 33 | 0 | 0.35 | flexibility |
| 14 | Sungkarat et al. | 2016 |  | 3 | 50 | 15 | 33 | 29.4 | 44.23 | 33 | -7.3 | 86.17 | flexibility |
