# Supplementary material for: Nonlinear dose-response of mind-body exercise in older adults with mild cognitive impairment: A systematic review and meta-analysis
Source: PLoS One. 2026 Sep 25;21(9):e0350867. doi: 10.1371/journal.pone.0350867 (PMC13614650; doi:10.1371/journal.pone.0350867)
Supplement: Supplementary file 2 [file pone.0350867.s003.docx]

Manuscript ID: PONE-D-26-24173

Title: Nonlinear Dose-Response of Mind-Body Exercise in Older Adults with Mild Cognitive Impairment: A Systematic Review and Meta-Analysis

Dear Editor,

We sincerely appreciate your professional comments and detailed suggestions which have greatly helped us improve the quality of our manuscript. We have carefully revised the manuscript in accordance with all the points raised. Below, we provide a point-by-point response to the editorial requests.

1、 **Responses to points 1–3 and 6–7:**

We have revised the manuscript format to comply with PLOS ONE’s style requirements. Specifically, we have removed funding information from the manuscript and included the Role of Funder statement in the cover letter as requested. We have also removed all figures from the main manuscript, uploaded each figure as a separate TIFF file, and retained the tables within the main manuscript while deleting the individual table files.

2、 **Responses to Data Availability Statement:**

In accordance with the minimal data set requirement, we have uploaded the dataset and the analysis code as supplementary files (code, data).

3、 **Responses to ethics statement and reference list:**

We have added an ethics statement to the Methods section as requested. The statement clarifies that, as a meta-analysis of previously published and anonymized data, this study did not require ethical approval or informed consent. Please see Page 8, Lines 141–144. We have carefully checked and verified all references in the list.

**4. Responses to Reviewer #2:**

Following Reviewer #2’s suggestions, we have thoroughly revised the Discussion section to further elaborate on the concept of the "nadir," reducing potential ambiguity, and have added a dedicated discussion on the potential underlying mechanisms. Please see Page 20–23, Lines 317–349 and 366–379.

We believe the manuscript has been substantially improved through these revisions and hope it now meets the standards for publication in PLOS ONE. Should you have any further questions or require additional modifications, please do not hesitate to let us know.

Thank you again for your time and consideration.

Yours sincerely

Yan-Fang Peng

(on behalf of all authors)
