# Supplementary material for: Nonlinear dose-response of mind-body exercise in older adults with mild cognitive impairment: A systematic review and meta-analysis
Source: PLoS One. 2026 Sep 25;21(9):e0350867. doi: 10.1371/journal.pone.0350867 (PMC13614650; doi:10.1371/journal.pone.0350867)
Supplement: Supplementary file 3 [file pone.0350867.s004.docx]

Response to reviewers

Manuscript ID: PONE-D-26-24173

Title: Nonlinear Dose-Response of Mind-Body Exercise in Older Adults with Mild Cognitive Impairment: A Systematic Review and Meta-Analysis

Dear Editor and Reviewers,

We sincerely thank the editor and the reviewer for the thoughtful and constructive evaluation of our manuscript. The reviewer's comments have substantially improved the rigor and clarity of our work. We have carefully revised the manuscript to address all concerns. Below, we provide a point-by-point response to each comment.

***Comments 1:*** *The clinical interpretation of the estimated 240 min/week nadir should be substantially tempered. The estimated effect at the reported nadir remains beneficial; thus, this point represents a relative minimum in the fitted curve rather than evidence of an ineffective or harmful dose. The authors should remove recommendations to “avoid” this dose and instead describe the finding as an exploratory nonlinear association requiring prospective confirmation.*

***Comments 2:*** *The dose-response findings should be explicitly described as study-level associations rather than individual-level causal effects. Exercise dose was not randomized across participants. The manuscript should acknowledge the potential for ecological/meta-regression confounding and avoid implying that changing an individual patient’s weekly exercise dose would necessarily produce the shape observed in the spline analysis.*

***Comments 4:*** *The concept of a “minimum effective dose” should be reconsidered. The threshold for statistical significance is not equal to the validated biological or clinically important dose threshold.*

1、 **Responses to** **comments 1, 2 and 4:**

We fully agree with the reviewer's astute observation and sincerely appreciate the opportunity to clarify our findings. We acknowledge that our original characterization of the 240 min/week nadir and minimum effective dose contained ambiguity. We have made the following revisions:

1） To reduce ambiguity, we have added the pooled effect estimate at this point (Hedges' g = 0.37; 95% CI: [0.19, 0.55]) corresponding to 240 min/week in the abstract (revised Abstract, Pag 2, liner: 10-21). We have also removed the inappropriate phrasing ‘avoid’ a weekly exercise dose of approximately 240 min/week throughout the manuscript (revised Discussion, Pag 21, liner: 323-324). Furthermore, we emphasize that the findings were not individual-level causal effects, and prospective validation of these findings is required prior to clinical application (revised Conclusion, Pag 23, liner: 360-364).
2） We agree that statistical significance (p < 0.05) does not equate to a validated biological or clinical dose threshold. We have removed all references to "minimum effective dose" and "minimum dose threshold" and replaced them with the following neutral description: "the dose at which the 95% confidence interval of the effect estimate first excludes the null value," with an explicit caveat that this is a statistical, not biological, threshold (revised Methods Pag 7, liner 100-101; Results sections Pag 16, liner: 232-235; Pag 17, liner: 248-250; Pag 18, liner: 268-271; and Discussion, Pag 21, liner: 322-325).

***Comments 3:*** *Please provide substantially greater detail regarding the statistical implementation of the restricted cubic spline analyses. Please provide the complete model specification, including weighting, working correlation structure, etc.*

2、 **Responses to comments 3:**

We thank the reviewer for this important suggestion. We note that the specific knot positions for each model were already reported in the Results section. However, we agree that the Methods section lacked a complete description of the knot placement strategy and full model specification.

The key revisions to the Methods (Data synthesis) section included 1) a detailed description of the Harrell percentile-based knot placement strategy; 2) a complete model specification, including the regression equation; 3) an explicit statement of the weighting scheme, which comprised an unweighted primary analysis and an inverse-variance weighted sensitivity analysis (results presented in a S2 Table); and 4) replacement of the “minimum effective dose” (revised Methods Pag 6-7, liner 82-104).

***Comments 5:*** *Please also correct internal inconsistencies and undertake careful language editing. For example, the abstract reports 27 RCTs including 2,034 participants, whereas the Results report 2,056 participants.*

**3、Responses to comments 5:**

We thank the reviewer for identifying this inconsistency and apologize for the confusion. We have corrected this discrepancy as follows:

2056 represents the total number of participants initially randomized/recruited across the 27 included RCTs. 2034 represents the number of participants included in the final data analyses. We have added the detailed information to mitigate ambiguity (revised Results, Pag 9, liner 147-148).

We hope that revisions adequately address the reviewer’s concerns. We are grateful for the opportunity to improve our manuscript and welcome any further suggestions.

Sincerely

Yan-Fang Peng

(on behalf of all authors)
