# Supplementary material for: Nonlinear dose-response of mind-body exercise in older adults with mild cognitive impairment: A systematic review and meta-analysis
Source: PLoS One. 2026 Sep 25;21(9):e0350867. doi: 10.1371/journal.pone.0350867 (PMC13614650; doi:10.1371/journal.pone.0350867)
Supplement: Peer review history

# Peer review: Decision Letter 0

**DOI:** 10.1371/journal.pone.0350867.r001

**Authors:** Ioannis Liampas

15 Jul 2026

Dear Dr. Peng,

Thank you for submitting your manuscript to PLOS ONE. After careful consideration, we feel that it has merit but does not fully meet PLOS ONE’s publication criteria as it currently stands. Therefore, we invite you to submit a revised version of the manuscript that addresses the points raised during the review process.

Please submit your revised manuscript by Aug 29 2026 11:59PM. If you will need more time than this to complete your revisions, please reply to this message or contact the journal office at plosone@plos.org. When you're ready to submit your revision, log on to https://www.editorialmanager.com/pone/ and select the 'Submissions Needing Revision' folder to locate your manuscript file.

- A letter that responds to each point raised by the academic editor and reviewer(s). You should upload this letter as a separate file labeled 'Response to Reviewers'.
- A marked-up copy of your manuscript that highlights changes made to the original version. You should upload this as a separate file labeled 'Revised Manuscript with Track Changes'.
- An unmarked version of your revised paper without tracked changes. You should upload this as a separate file labeled 'Manuscript'.

If you would like to make changes to your financial disclosure, please include your updated statement in your cover letter. Guidelines for resubmitting your figure files are available below the reviewer comments at the end of this letter.

If applicable, we recommend that you deposit your laboratory protocols in protocols.io to enhance the reproducibility of your results. Protocols.io assigns your protocol its own identifier (DOI) so that it can be cited independently in the future. For instructions see: https://journals.plos.org/plosone/s/submission-guidelines#loc-laboratory-protocols. Additionally, PLOS ONE offers an option for publishing peer-reviewed Lab Protocol articles, which describe protocols hosted on protocols.io. Read more information on sharing protocols at https://plos.org/protocols?utm_medium=editorial-email&utm_source=authorletters&utm_campaign=protocols.

As the corresponding author, your ORCID iD is verified in the submission system and will appear in the published article. PLOS supports the use of ORCID, and we encourage all coauthors to register for an ORCID iD and use it as well. Please encourage your coauthors to verify their ORCID iD within the submission system before final acceptance, as unverified ORCID iDs will not appear in the published article. *Only*  the individual author can complete the verification step; PLOS staff *cannot*  verify ORCID iDs on behalf of authors.

We look forward to receiving your revised manuscript.

Kind regards,

Ioannis Liampas, MD. PhD

Academic Editor

PLOS One

Journal Requirements:

When submitting your revision, we need you to address these additional requirements.

1.Please ensure that your manuscript meets PLOS ONE's style requirements, including those for file naming. The PLOS ONE style templates can be found at

https://journals.plos.org/plosone/s/file?id=wjVg/PLOSOne_formatting_sample_main_body.pdf and

https://journals.plos.org/plosone/s/file?id=ba62/PLOSOne_formatting_sample_title_authors_affiliations.pdf

2. Please note that funding information should not appear in any section or other areas of your manuscript. We will only publish funding information present in the Funding Statement section of the online submission form. Please remove any funding-related text from the manuscript.

3. Thank you for stating the following financial disclosure:

“This project is supported by the Zhejiang office of Philosophy and Social Science Planning (grant no. 23NDJC203YB), Hubei Provincial Department of Education (grant no. B2023210; 24Y113)”

Please state what role the funders took in the study.  If the funders had no role, please state: "The funders had no role in study design, data collection and analysis, decision to publish, or preparation of the manuscript."

If this statement is not correct you must amend it as needed.

Please include this amended Role of Funder statement in your cover letter; we will change the online submission form on your behalf.

4. We note that your Data Availability Statement is currently as follows: “All relevant data are within the manuscript and its supporting information files. The

included studies are publicly available published literature, which can be retrieved from the corresponding databases. No raw experimental data were generated in this systematic review.”

Please confirm at this time whether or not your submission contains all raw data required to replicate the results of your study. Authors must share the “minimal data set” for their submission. PLOS defines the minimal data set to consist of the data required to replicate all study findings reported in the article, as well as related metadata and methods (https://journals.plos.org/plosone/s/data-availability#loc-minimal-data-set-definition).

For example, authors should submit the following data:

- The values behind the means, standard deviations and other measures reported;

- The values used to build graphs;

- The points extracted from images for analysis.

Authors do not need to submit their entire data set if only a portion of the data was used in the reported study.

If your submission does not contain these data, please either upload them as Supporting Information files or deposit them to a stable, public repository and provide us with the relevant URLs, DOIs, or accession numbers. For a list of recommended repositories, please see https://journals.plos.org/plosone/s/recommended-repositories.

If there are ethical or legal restrictions on sharing a de-identified data set, please explain them in detail (e.g., data contain potentially sensitive information, data are owned by a third-party organization, etc.) and who has imposed them (e.g., an ethics committee). Please also provide contact information for a data access committee, ethics committee, or other institutional body to which data requests may be sent. If data are owned by a third party, please indicate how others may request data access.

5. Please include your full ethics statement in the ‘Methods’ section of your manuscript file. In your statement, please include the full name of the IRB or ethics committee who approved or waived your study, as well as whether or not you obtained informed written or verbal consent. If consent was waived for your study, please include this information in your statement as well.

6. Please remove your figures from within your manuscript file, leaving only the individual TIFF/EPS image files, uploaded separately. These will be automatically included in the reviewers’ PDF.

7. Please include your tables as part of your main manuscript and remove the individual files. Please note that supplementary tables (should remain/ be uploaded) as separate "supporting information" files.

8. If the reviewer comments include a recommendation to cite specific previously published works, please review and evaluate these publications to determine whether they are relevant and should be cited. There is no requirement to cite these works unless the editor has indicated otherwise.

9. Please review your reference list to ensure that it is complete and correct. If you have cited papers that have been retracted, please include the rationale for doing so in the manuscript text, or remove these references and replace them with relevant current references. Any changes to the reference list should be mentioned in the rebuttal letter that accompanies your revised manuscript. If you need to cite a retracted article, indicate the article’s retracted status in the References list and also include a citation and full reference for the retraction notice.

[Note: HTML markup is below. Please do not edit.]

Reviewers' comments:

Reviewer's Responses to Questions

**Comments to the Author**

1. Is the manuscript technically sound, and do the data support the conclusions?

Reviewer #1: Yes

Reviewer #2: Yes

**********

2. Has the statistical analysis been performed appropriately and rigorously?

Reviewer #1: Yes

Reviewer #2: Yes

**********

3. Have the authors made all data underlying the findings in their manuscript fully available?

The PLOS Data policy

Reviewer #1: Yes

Reviewer #2: Yes

**********

4. Is the manuscript presented in an intelligible fashion and written in standard English?

Reviewer #1: Yes

Reviewer #2: Yes

**********

Reviewer #1: This is an important perspective and will be of interest for the readership of the journal, as it sheds light on non-pharmacological interventions against MCI which are largely neglected in clinical algorithms. The methods are sound. The main clinical implications are listed.

Reviewer #2: Overall Recommendation: Minor revision

This is a well-designed, rigorously conducted, and analytically sophisticated systematic review and meta-analysis. Its core strength lies in going beyond the simple question of "whether" mind-body exercise is effective, to address the more clinically relevant question of "how" it works—by characterizing domain-specific dose-response relationships and, more importantly, quantifying the interactions among key exercise prescription parameters. This work provides a critical scientific foundation for precision exercise prescription in the field of cognitive impairment. The study involves a substantial body of work, employs state-of-the-art statistical methods, and demonstrates significant potential for clinical translation.

Minor Suggestions

1. The interpretation of the "nadir" requires greater caution.

The manuscript identifies 240 minutes/week as the nadir of benefit, implying that cognitive improvement is minimal at this dose, and recommends avoiding it. This is a strong conclusion that could be misinterpreted by clinicians as indicating a "harmful dose" or a "completely ineffective dose."

Suggestion: In the Discussion section, we recommend explicitly clarifying that this nadir represents the relative lowest point of effect observed within the effective dose range (90–450 minutes/week), and should not be construed as a toxic or ineffective threshold. At this dose, cognitive function still demonstrates measurable improvement (Hedges' *g* = 0.37), albeit with a smaller effect size compared to lower or higher weekly doses. Such clarification is essential to prevent potential misunderstanding and to ensure appropriate clinical application of these findings.

2. The mechanistic basis for the observed interaction effect warrants further discussion.

The study found that intervention duration positively moderates the effect of weekly dose (β = 0.012). The authors interpret this as "longer duration yields greater benefits," but the underlying mechanisms are not thoroughly explored.

Suggestion: We suggest incorporating a discussion grounded in neuroplasticity theory to enrich the mechanistic interpretation. For example, longer intervention periods may facilitate more sustained neurobiological changes, such as prolonged upregulation of brain-derived neurotrophic factor (BDNF), improved cerebrovascular health, or the consolidation of exercise adherence and behavioral habits. These cumulative adaptations could render longer-term, lower-dose regimens ultimately effective, whereas shorter, higher-dose interventions may fail to produce lasting benefits due to insufficient duration of exposure. Such a perspective would strengthen the conceptual framework and enhance the clinical interpretability of the findings.

**********

what does this mean?). If published, this will include your full peer review and any attached files.

If you choose “no”, your identity will remain anonymous but your review may still be made public.

**Do you want your identity to be public for this peer review?**  For information about this choice, including consent withdrawal, please see our Privacy Policy

Reviewer #1: No

Reviewer #2: No

**********

[NOTE: If reviewer comments were submitted as an attachment file, they will be attached to this email and accessible via the submission site. Please log into your account, locate the manuscript record, and check for the action link "View Attachments". If this link does not appear, there are no attachment files.]

To ensure your figures meet our technical requirements, please review our figure guidelines: https://journals.plos.org/plosone/s/figures

You may also use PLOS’s free figure tool, NAAS, to help you prepare publication quality figures: https://journals.plos.org/plosone/s/figures#loc-tools-for-figure-preparation.

NAAS will assess whether your figures meet our technical requirements by comparing each figure against our figure specifications.

# Author comment: Author response to Decision Letter 1

**DOI:** 10.1371/journal.pone.0350867.r002

1 Aug 2026

Dear Editor,

We sincerely appreciate your professional comments and detailed suggestions which have greatly helped us improve the quality of our manuscript. We have carefully revised the manuscript in accordance with all the points raised. Below, we provide a point-by-point response to the editorial requests.

1、 Responses to points 1–3 and 6–7:

We have revised the manuscript format to comply with PLOS ONE’s style requirements. Specifically, we have removed funding information from the manuscript and included the Role of Funder statement in the cover letter as requested. We have also removed all figures from the main manuscript, uploaded each figure as a separate TIFF file, and retained the tables within the main manuscript while deleting the individual table files.

2、 Responses to Data Availability Statement:

In accordance with the minimal data set requirement, we have uploaded the dataset and the analysis code as supplementary files (code, data).

3、 Responses to ethics statement and reference list:

We have added an ethics statement to the Methods section as requested. The statement clarifies that, as a meta-analysis of previously published and anonymized data, this study did not require ethical approval or informed consent. Please see Page 8, Lines 141–144. We have carefully checked and verified all references in the list.

4. Responses to Reviewer #2:

Following Reviewer #2’s suggestions, we have thoroughly revised the Discussion section to further elaborate on the concept of the "nadir," reducing potential ambiguity, and have added a dedicated discussion on the potential underlying mechanisms. Please see Page 20–23, Lines 317–349 and 366–379.

We believe the manuscript has been substantially improved through these revisions and hope it now meets the standards for publication in PLOS ONE. Should you have any further questions or require additional modifications, please do not hesitate to let us know.

Thank you again for your time and consideration.

Attachment Submitted filename: Response to reviewers.docx

# Editor report: Decision Letter 1

**DOI:** 10.1371/journal.pone.0350867.r003

**Authors:** Ioannis Liampas

10 Aug 2026

Dear Dr. Peng,

Thank you for submitting your manuscript to PLOS ONE. After careful consideration, we feel that it has merit but does not fully meet PLOS ONE’s publication criteria as it currently stands. Therefore, we invite you to submit a revised version of the manuscript that addresses the points raised during the review process.

Please submit your revised manuscript by Sep 09 2026 11:59PM. If you will need more time than this to complete your revisions, please reply to this message or contact the journal office at plosone@plos.org. When you're ready to submit your revision, log on to https://www.editorialmanager.com/pone/ and select the 'Submissions Needing Revision' folder to locate your manuscript file.

- A letter that responds to each point raised by the academic editor and reviewer(s). You should upload this letter as a separate file labeled 'Response to Reviewers'.
- A marked-up copy of your manuscript that highlights changes made to the original version. You should upload this as a separate file labeled 'Revised Manuscript with Track Changes'.
- An unmarked version of your revised paper without tracked changes. You should upload this as a separate file labeled 'Manuscript'.

If you would like to make changes to your financial disclosure, please include your updated statement in your cover letter. Guidelines for resubmitting your figure files are available below the reviewer comments at the end of this letter.

If applicable, we recommend that you deposit your laboratory protocols in protocols.io to enhance the reproducibility of your results. Protocols.io assigns your protocol its own identifier (DOI) so that it can be cited independently in the future. For instructions see: https://journals.plos.org/plosone/s/submission-guidelines#loc-laboratory-protocols. Additionally, PLOS ONE offers an option for publishing peer-reviewed Lab Protocol articles, which describe protocols hosted on protocols.io. Read more information on sharing protocols at https://plos.org/protocols?utm_medium=editorial-email&utm_source=authorletters&utm_campaign=protocols.

As the corresponding author, your ORCID iD is verified in the submission system and will appear in the published article. PLOS supports the use of ORCID, and we encourage all coauthors to register for an ORCID iD and use it as well. Please encourage your coauthors to verify their ORCID iD within the submission system before final acceptance, as unverified ORCID iDs will not appear in the published article. *Only*  the individual author can complete the verification step; PLOS staff *cannot*  verify ORCID iDs on behalf of authors.

We look forward to receiving your revised manuscript.

Kind regards,

Ioannis Liampas, MD. PhD

Academic Editor

PLOS One

Journal Requirements:

If the reviewer comments include a recommendation to cite specific previously published works, please review and evaluate these publications to determine whether they are relevant and should be cited. There is no requirement to cite these works unless the editor has indicated otherwise.

Please review your reference list to ensure that it is complete and correct. If you have cited papers that have been retracted, please include the rationale for doing so in the manuscript text, or remove these references and replace them with relevant current references. Any changes to the reference list should be mentioned in the rebuttal letter that accompanies your revised manuscript. If you need to cite a retracted article, indicate the article’s retracted status in the References list and also include a citation and full reference for the retraction notice.

Additional Editor Comments:

The article is potentially suitable for publication. The following interpretative issues should be addressed before acceptance.

1. The clinical interpretation of the estimated 240 min/week nadir should be substantially tempered. The estimated effect at the reported nadir remains beneficial; thus, this point represents a relative minimum in the fitted curve rather than evidence of an ineffective or harmful dose. The authors should remove recommendations to “avoid” this dose and instead describe the finding as an exploratory nonlinear association requiring prospective confirmation.

2. The dose-response findings should be explicitly described as study-level associations rather than individual-level causal effects. Exercise dose was not randomized across participants. The manuscript should acknowledge the potential for ecological/meta-regression confounding and avoid implying that changing an individual patient’s weekly exercise dose would necessarily produce the shape observed in the spline analysis.

3. Please provide substantially greater detail regarding the statistical implementation of the restricted cubic spline analyses. Please provide the complete model specification, including weighting, working correlation structure, etc.

4. The concept of a “minimum effective dose” should be reconsidered. The threshold for statistical significance is not equal to the validated biological or clinically important dose threshold.

5. Please also correct internal inconsistencies and undertake careful language editing. For example, the abstract reports 27 RCTs including 2,034 participants, whereas the Results report 2,056 participants.

[Note: HTML markup is below. Please do not edit.]

[NOTE: If reviewer comments were submitted as an attachment file, they will be attached to this email and accessible via the submission site. Please log into your account, locate the manuscript record, and check for the action link "View Attachments". If this link does not appear, there are no attachment files.]

To ensure your figures meet our technical requirements, please review our figure guidelines: https://journals.plos.org/plosone/s/figures

You may also use PLOS’s free figure tool, NAAS, to help you prepare publication quality figures: https://journals.plos.org/plosone/s/figures#loc-tools-for-figure-preparation.

NAAS will assess whether your figures meet our technical requirements by comparing each figure against our figure specifications.

# Author comment: Author response to Decision Letter 2

**DOI:** 10.1371/journal.pone.0350867.r004

3 Sep 2026

Manuscript ID: PONE-D-26-24173

Title: Nonlinear Dose-Response of Mind-Body Exercise in Older Adults with Mild Cognitive Impairment: A Systematic Review and Meta-Analysis

Dear Editor and Reviewers,

We sincerely thank the editor and the reviewer for the thoughtful and constructive evaluation of our manuscript. The reviewer's comments have substantially improved the rigor and clarity of our work. We have carefully revised the manuscript to address all concerns. Below, we provide a point-by-point response to each comment.

Comments 1: The clinical interpretation of the estimated 240 min/week nadir should be substantially tempered. The estimated effect at the reported nadir remains beneficial; thus, this point represents a relative minimum in the fitted curve rather than evidence of an ineffective or harmful dose. The authors should remove recommendations to “avoid” this dose and instead describe the finding as an exploratory nonlinear association requiring prospective confirmation.

Comments 2: The dose-response findings should be explicitly described as study-level associations rather than individual-level causal effects. Exercise dose was not randomized across participants. The manuscript should acknowledge the potential for ecological/meta-regression confounding and avoid implying that changing an individual patient’s weekly exercise dose would necessarily produce the shape observed in the spline analysis.

Comments 4: The concept of a “minimum effective dose” should be reconsidered. The threshold for statistical significance is not equal to the validated biological or clinically important dose threshold.

1、 Responses to comments 1, 2 and 4:

We fully agree with the reviewer's astute observation and sincerely appreciate the opportunity to clarify our findings. We acknowledge that our original characterization of the 240 min/week nadir and minimum effective dose contained ambiguity. We have made the following revisions:

1） To reduce ambiguity, we have added the pooled effect estimate at this point (Hedges' g = 0.37; 95% CI: [0.19, 0.55]) corresponding to 240 min/week in the abstract (revised Abstract, Pag 2, liner: 10-21). We have also removed the inappropriate phrasing ‘avoid’ a weekly exercise dose of approximately 240 min/week throughout the manuscript (revised Discussion, Pag 21, liner: 323-324). Furthermore, we emphasize that the findings were not individual-level causal effects, and prospective validation of these findings is required prior to clinical application (revised Conclusion, Pag 23, liner: 360-364).

2） We agree that statistical significance (p < 0.05) does not equate to a validated biological or clinical dose threshold. We have removed all references to "minimum effective dose" and "minimum dose threshold" and replaced them with the following neutral description: "the dose at which the 95% confidence interval of the effect estimate first excludes the null value," with an explicit caveat that this is a statistical, not biological, threshold (revised Methods Pag 7, liner 100-101; Results sections Pag 16, liner: 232-235; Pag 17, liner: 248-250; Pag 18, liner: 268-271; and Discussion, Pag 21, liner: 322-325).

Comments 3: Please provide substantially greater detail regarding the statistical implementation of the restricted cubic spline analyses. Please provide the complete model specification, including weighting, working correlation structure, etc.

2、 Responses to comments 3:

We thank the reviewer for this important suggestion. We note that the specific knot positions for each model were already reported in the Results section. However, we agree that the Methods section lacked a complete description of the knot placement strategy and full model specification.

The key revisions to the Methods (Data synthesis) section included 1) a detailed description of the Harrell percentile-based knot placement strategy; 2) a complete model specification, including the regression equation; 3) an explicit statement of the weighting scheme, which comprised an unweighted primary analysis and an inverse-variance weighted sensitivity analysis (results presented in a S2 Table); and 4) replacement of the “minimum effective dose” (revised Methods Pag 6-7, liner 82-104).

Comments 5: Please also correct internal inconsistencies and undertake careful language editing. For example, the abstract reports 27 RCTs including 2,034 participants, whereas the Results report 2,056 participants.

3、Responses to comments 5:

We thank the reviewer for identifying this inconsistency and apologize for the confusion. We have corrected this discrepancy as follows:

2056 represents the total number of participants initially randomized/recruited across the 27 included RCTs. 2034 represents the number of participants included in the final data analyses. We have added the detailed information to mitigate ambiguity (revised Results, Pag 9, liner 147-148).

We hope that revisions adequately address the reviewer’s concerns. We are grateful for the opportunity to improve our manuscript and welcome any further suggestions.

Sincerely

Yan-Fang Peng

(on behalf of all authors)

Attachment Submitted filename: Response_to_reviewers_auresp_2.docx

# Editor report: Decision Letter 2

**DOI:** 10.1371/journal.pone.0350867.r005

**Authors:** Ioannis Liampas

6 Sep 2026

<p>Nonlinear Dose-Response of Mind-Body Exercise in Older Adults with Mild Cognitive Impairment: A Systematic Review and Meta-Analysis

PONE-D-26-24173R2

Dear Dr. Peng,

We’re pleased to inform you that your manuscript has been judged scientifically suitable for publication and will be formally accepted for publication once it meets all outstanding technical requirements.

Within one week, you’ll receive an e-mail detailing the required amendments. When these have been addressed, you’ll receive a formal acceptance letter and your manuscript will be scheduled for publication.

An invoice will be generated when your article is formally accepted. Please note, if your institution has a publishing partnership with PLOS and your article meets the relevant criteria, all or part of your publication costs will be covered. Please make sure your user information is up-to-date by logging into Editorial Manager at Editorial Manager® and clicking the ‘Update My Information' link at the top of the page. For questions related to billing, please contact billing support.

If your institution or institutions have a press office, please notify them about your upcoming paper to help maximize its impact. If they’ll be preparing press materials, please inform our press team as soon as possible -- no later than 48 hours after receiving the formal acceptance. Your manuscript will remain under strict press embargo until 2 pm Eastern Time on the date of publication. For more information, please contact onepress@plos.org.

Kind regards,

Ioannis Liampas, MD. PhD

Academic Editor

PLOS One

Additional Editor Comments (optional):

Reviewers' comments:

# Editor report: Acceptance letter

**DOI:** 10.1371/journal.pone.0350867.r006

**Authors:** Ioannis Liampas

PONE-D-26-24173R2

PLOS One

Dear Dr. Peng,

I'm pleased to inform you that your manuscript has been deemed suitable for publication in PLOS One. Congratulations! Your manuscript is now being handed over to our production team.

At this stage, our production department will prepare your paper for publication. This includes ensuring the following:

* All references, tables, and figures are properly cited

* All relevant supporting information is included in the manuscript submission,

* There are no issues that prevent the paper from being properly typeset

You will receive further instructions from the production team, including instructions on how to review your proof when it is ready. Please keep in mind that we are working through a large volume of accepted articles, so please give us a few days to review your paper and let you know the next and final steps.

Lastly, if your institution or institutions have a press office, please let them know about your upcoming paper now to help maximize its impact. If they'll be preparing press materials, please inform our press team within the next 48 hours. Your manuscript will remain under strict press embargo until 2 pm Eastern Time on the date of publication. For more information, please contact onepress@plos.org.

You will receive an invoice from PLOS for your publication fee after your manuscript has reached the completed accept phase. If you receive an email requesting payment before acceptance or for any other service, this may be a phishing scheme. Learn how to identify phishing emails and protect your accounts at https://explore.plos.org/phishing.

If we can help with anything else, please email us at customercare@plos.org.

Thank you for submitting your work to PLOS One and supporting open access.

Kind regards,

PLOS One Editorial Office Staff

on behalf of

Dr. Ioannis Liampas

Academic Editor

PLOS One
